# Behavioral alignment in social networks

**DOI:** 10.1093/nsr/nwaf403

**Published:** 2025-09-19

**Authors:** Yu Xia, Alex McAvoy, Qi Su

**Affiliations:** School of Automation and Intelligent Sensing, Shanghai Jiao Tong University, Shanghai 200240, China; Key Laboratory of System Control and Information Processing, Ministry of Education of China, Shanghai 200240, China; Shanghai Key Laboratory of Perception and Control in Industrial Network Systems, Shanghai 200240, China; School of Data Science and Society, University of North Carolina at Chapel Hill, Chapel Hill, NC 27599, USA; Department of Mathematics, University of North Carolina at Chapel Hill, Chapel Hill, NC 27599, USA; School of Automation and Intelligent Sensing, Shanghai Jiao Tong University, Shanghai 200240, China; Key Laboratory of System Control and Information Processing, Ministry of Education of China, Shanghai 200240, China; Shanghai Key Laboratory of Perception and Control in Industrial Network Systems, Shanghai 200240, China

**Keywords:** game theory, complex network, best response, evolutionary dynamics

## Abstract

The orderly behaviors observed in large-scale groups, such as fish schooling and the organized movement of crowds, are both ubiquitous and essential for the survival and stability of these systems. Understanding how such complex collective behaviors emerge from simple local interactions and behavioral adjustments is a significant scientific challenge. Historically, research has predominantly focused on imitation and social learning, where individuals adopt the strategies of more successful peers to refine their behavior. However, in recent years, an alternative learning approach based on self-exploration and introspective learning has garnered increasing attention. In this paradigm, individuals assess their own circumstances and select strategies that best align with their specific conditions. Two examples are coordination and anti-coordination, where individuals align with and diverge from the local majority, respectively. In this study, we analyze networked systems of coordinating and anti-coordinating individuals, exploring the combined effects of system dynamics, network structure and behavioral patterns. We address several practical questions, including the number of equilibria, their characteristics, the equilibrium time and the resilience of the system. We find that the number of equilibrium states can be extremely large, even increasing exponentially with minor alterations to the network structure. Moreover, the network structure has a significant impact on the average equilibrium time. Despite the complexity of these findings, we find that variations can be captured by a single, simple network characteristic (the average path length), which we illustrate in both synthetic and empirical networks.

## Teaser

This work uncovers how local interactions among agents generate system-level patterns, with average path length emerging as a universal predictor of collective dynamics.

## INTRODUCTION

The nature of individual decision-making processes can profoundly influence evolutionary outcomes in large-scale systems, including many populations found in social, biological, economic and ecological contexts [1–4]. A long-standing question across these domains is how simple local interactions collectively give rise to organized, system-level behavior, such as consensus, polarization or innovation diffusion [5,6]. Addressing this question requires understanding both the behavioral rules individuals follow and how these rules interact with the underlying network structures. Imitation is an important and well-studied social learning mechanism, in which individuals copy the behavior of those who are more successful or authoritative [7,8]. When combined with social network structure, imitation can drive the evolution of prosocial traits like cooperation [9], behaviors that otherwise would not be chosen by perfectly rational agents. A drawback of imitation is that it requires individuals to have extensive information, including the behaviors of their direct neighbors as well as the neighbors of those neighbors, both to assess the behaviors and imitate those they evaluate as being successful [10].

Conversely, coordination and anti-coordination as decision-making approaches allow individuals to independently seek the most beneficial strategy based on analyzing information from interaction partners. These forms of decision-making involve the ability to self-assess, enabling a deeper level of autonomy and robustness not achievable through imitation alone. Coordination, where individuals conform to the majority, and anti-coordination, where individuals diverge from the majority, can also be found in many real-life scenarios. Both are fundamental behavioral mechanisms consistently observed across diverse empirical systems. Examples of coordination include the formation of driving conventions, where people drive on the same side of the road, and the choice of language within multilingual communities, where individuals tend to use the language adopted by most people [11,12]. On the other hand, instances of anti-coordination include auction strategies, where some bidders choose less popular items in order to avoid competition, and problems of resource allocation, where variation in resource consumption and use can mitigate over-exploitation [13,14].

It is known that systems following either coordinating or anti-coordinating patterns must converge to an equilibrium state under asynchronous activation [15]. In fact, convergence to an equilibrium in such systems occurs in a finite number of strategy updates [16]. However, convergence properties of the system are sensitive to the initial strategy composition and behavioral switching threshold [17–20]. These studies focus mainly on convergence itself, leaving some critical aspects yet to be explored. Dynamical systems often exhibit a multitude of equilibrium states, a phenomenon observed in epidemiology, network synchronization and the evolution of ecosystems [21–24]. The variety of equilibrium states can have profound implications for a system. For example, shallow lake systems have two equilibrium states: clear and turbid. The clear state is rich in submerged vegetation, while in the turbid state, animal diversity is lost and algae is reduced [25]. It is natural, then, to be concerned with a system’s equilibrium probability distribution and equilibration time. For instance, in evolutionary game theory, fixation probabilities and times are important indicators of whether cooperation is favored [26–30].

The physical structure of a system profoundly influences the outcomes of evolution [31–36]. Degree distribution heterogeneity and the clustering coefficient represent two important measures of heterogeneity in networked systems [37,38]. In many networks, there exist highly connected nodes, called hubs, and nodes tend to form tightly knit groups in networks with high clustering coefficients. Both of these properties greatly influence the dynamical characteristics of networked systems. For example, heterogeneity in the interaction frequency between pairs of individuals can facilitate the evolution of cooperation [39]. In disease transmission models, heterogeneous networks might see rapid outbreaks of epidemics because hubs can accelerate disease spread, and networks with high clustering coefficients may lead to intense local outbreaks, as infections spread rapidly within closely interconnected groups [40–42]. In quite a different setting, heterogeneous networks are strongly resilient to random network attacks, but remain vulnerable to targeted strategies, and networks with high clustering coefficients possess a higher percolation threshold, which increases the cost of attacks [43,44]. Hence, thoroughly analyzing the impact of network structure is essential for advancing our understanding of network dynamics.

In this paper, we investigate the dynamics of coordination and anti-coordination systems, including key properties such as the number of equilibrium states, equilibration time, probability distributions and system resilience. Our analysis reveals that structural features of the underlying network, such as the degree distribution and clustering coefficient, strongly affect the number of equilibrium states. Notably, even small changes in network topology can lead to orders-of-magnitude variations in the number of equilibria. We further show that both the number of equilibrium states and the time required to reach equilibrium are governed by a simple topological measure: the average path length. As the average path length increases, coordination systems tend to equilibrate more slowly, while anti-coordination systems tend to equilibrate more rapidly. Furthermore, we validate these theoretical findings using twenty empirical networks spanning diverse domains, sizes and densities. These include the karate club friendship network in a university setting [45], interaction patterns among Mexican political elites [46], a Facebook friendship network within a computer company [47] and social associations among wild Grévy’s zebras [48].

## RESULTS

### Model

We consider a population of *N* individuals, denoted by $\mathcal {N} = \lbrace 1,2,\dots , N\rbrace$. The spatial structure of the population is represented by a network with *N* nodes, in which each individual occupies exactly one node, and edges represent connections between individuals. For nodes *i* and *j*, we denote by $k_{ij}$ the edge weight between *i* and *j*, which is 1 if there is an edge between *i* and *j* and 0 otherwise. Every individual has one of two strategies, *A* or *B*, and plays a game with each neighbor. When both players adopt strategy *A*, they both get a payoff *a*. Similarly, when both players employ strategy *B*, they both receive a payoff *d*. If one player chooses strategy *A* and the other uses strategy *B*, the *A* individual gets *b* and the *B* individual gets *c*.

Two classes of games are of immediate interest. The first is a coordination game, in which both players prefer to use the same strategy rather than different strategies. This class of games is characterized by the inequalities $a>c$ and $d>b$. There are three Nash equilibria in a coordination game: two pure Nash equilibria at $(A,A)$ and $(B,B)$, as well as a mixed equilibrium in which both players use *A* with probability $\tau =(d-b) /(a-b-c+d)$. The second is an anti-coordination game, in which both players would prefer to use opposite strategies. This class of games is characterized by $c>a$ and $b>d$. Here, both $(A,B)$ and $(B,A)$ are Nash equilibria, as is the strategy in which both players use *A* with probability $\tau$. In the main text, we focus our attention on *pure* coordination and anti-coordination games, which means that $a=d$ for the former and $b=c$ for the latter. Thus, for each game, both pure-strategy Nash equilibria are Pareto efficient and the players are concerned only with coordinating or not. (In the online supplementary material, we consider examples where this assumption does not necessarily hold.)

In both games, every player is assigned an aggregate score obtained by accumulating the payoffs from all pairwise interactions. Let $s_{i}(t)$ be individual *i*’s strategy at time *t*, where $s_{i}(t) =1$ represents strategy *A* and $s_{i}(t) =0$ represents strategy *B*. The accumulated payoffs to individual *i* when *i* uses strategies *A* and *B*, respectively, at time *t* are


(1a) \begin{eqnarray*}
u_{i}^{A}(t) = \sum\limits_{{j \in \mathcal {N}}} k_{ij} [a s_{j}(t) + b (1-s_{j}(t))],
\end{eqnarray*}

(1b) \begin{eqnarray*}
u_{i}^{B}(t) = \sum\limits_{{j \in \mathcal {N}}} k_{ij}[ cs_{j}(t) + d (1-s_{j}(t))] .
\end{eqnarray*}

In each step, a random individual, *i*, is chosen uniformly from the system to update its strategy. Under best-response dynamics [49], this individual evaluates strategies *A* and *B*, adopting the one yielding the greater total payoff (or maintaining the current strategy if both yield the same payoff). If *i* is chosen at time *t* for a strategy revision then, from Equations (1), we have


(2) \begin{eqnarray*}
u_{i}^{A}\!-\!u_{i}^{B} &=& \sum\limits_{j \in \mathcal {N}} k_{ij}[a s_j \!+\! b (1\!-\!s_j) - c s_j - d (1\!-\!s_j) ] \\
&=& (a-b-c+d) n_i - (d-b) k_i,
\end{eqnarray*}

where $n_i = \sum _{j \in \mathcal {N}} k_{ij} s_j$ denotes the number of *i*’s neighbors using *A*, and $k_i = \sum _{j \in \mathcal {N}} k_{ij}$ is the total number of neighbors of *i* (i.e. the degree of *i*).

In a coordination game, $a-b-c+d>0$. Thus, under best-response dynamics, Equation (2) gives


(3) \begin{eqnarray*}
s_i (t+1) = \left\lbrace \begin{array}{@{}l@{\quad }l@{}}1, & n_i > \tau k_i , \\
s_i (t), & n_i = \tau k_i, \\
0, & n_i < \tau k_i , \end{array}\right.
\end{eqnarray*}

where, again, $\tau =(d-b) /(a-b-c+d)$, which coincides with the probability of playing *A* in the mixed-strategy Nash equilibrium. On the other hand, $a-b-c+d<0$ in an anti-coordination game, which yields a next-round action of


(4) \begin{eqnarray*}
s_i (t+1) = \left\lbrace \begin{array}{@{}l@{\quad }l@{}}1, & n_i < \tau k_i, \\
s_i (t), & n_i = \tau k_i, \\
0, & n_i > \tau k_i. \end{array}\right.
\end{eqnarray*}

**Figure 1.**
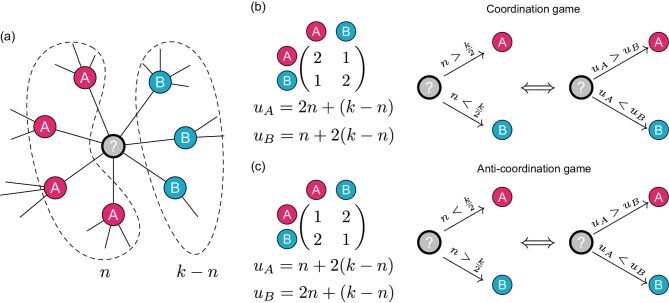
Strategy evolution under coordination and anti-coordination decision-making patterns. (a) The structure is described by a network, where each individual (node) adopts either strategy *A* or *B* to interact with neighbors. An individual (marked by ‘?’) is chosen to update its strategy to maximize payoff, interacting with *n* individuals using strategy *A* and $k-n$ individuals using strategy *B*. (b) An example of a coordination game, where the chosen individual receives a higher payoff when adopting the strategy used by the majority of his neighbors. For this payoff matrix, taking strategy *A* used by the majority brings a higher payoff than taking *B* whenever $n>k/2$. The individual opts for strategy *B* when $n<k/2$. The individual maintains its current strategy if $n=k/2$, which is equivalent to $u^{A} = u^{B}$. (c) An example of an anti-coordination game. When $n > k/2$, adopting strategy *B* yields a higher payoff. If $n < k/2$, strategy *A* leads to a higher payoff. The payoffs are the same when $n = k/2$.

Figure 1 shows examples of coordination and anti-coordination games, as well as switching thresholds under best-response dynamics.

It is worth noting what best-response dynamics would lead to for other classes of games, such as social dilemmas. In a prisoner’s dilemma, which is defined by $c>a>d>b$, we have $d-b>0$, but $a-b-c+d$ can be positive, negative or zero. However, in all three cases, the payoff difference in Equation (2) is negative, which means that a player’s best response is always *B*. This finding is not surprising, given that *B* is a dominant action in a prisoner’s dilemma and will always be used by a rational agent. However, there are also weaker social dilemmas such as snowdrift games, which are defined by $c>a>b>d$. This, in fact, is an example of an anti-coordination game (just not a *pure* anti-coordination game). For a snowdrift game, we have $\tau \in (0,1)$; in particular, unlike in a prisoner’s dilemma, the best response is non-trivial (Equation (4)).

A configuration of *A* and *B* is defined as an equilibrium state if, under the update rule in Equation (3) or (4), each individual’s prescribed next action matches their current action. A state $\mathbf {s}^{\ast }=(s_1^{\ast },\dots ,s_N^{\ast })$ is an equilibrium if $u_i(\mathbf {s}^{\ast })=\max \lbrace u_i^A(\mathbf {s}^{\ast }) ,u_i^B(\mathbf {s}^{\ast })\rbrace$ for all $i\in \mathcal {N}$, where $u_i(\mathbf {s}^{\ast })$ denotes player *i*’s payoff in state $\mathbf {s}^{\ast }$, and $u_i^X(\mathbf {s}^{\ast })$ ($X\in \lbrace A,B\rbrace$) is player *i*’s payoff when the other players use $s_j^{\ast }$  $(j\ne i)$ and *i* adopts strategy *X*. Consequently, no individual has the incentive to change strategy in an equilibrium state and $s_i^{\ast }(t+1) = s_i^{\ast }(t)$ for all $i \in \mathcal {N}$. We first explore the number of equilibrium states on representative networks and conduct a quantitative analysis of a class of networks to provide intuition about the interplay between network structure and evolutionary dynamics. Next, we investigate the absorption capacity of the equilibrium states and the average time required to reach them. Here, we define the absorption capacity of a given equilibrium state as the probability that the system, starting from a random initial configuration, eventually reaches that state. Finally, we examine the robustness of various network structures.

### The number of equilibrium states

We first investigate the impact of closed triads, a widely studied network property, on evolutionary outcomes. Closed triads, commonly found in empirical networks [52–54], indicate that two individuals connected to a common third party are likely to connect to each other as well. The prevalence of closed triads is quantified by the (global) clustering coefficient,


(5) \begin{eqnarray*}
\mathcal {C} = \frac{\sum _{i,j,k \in \mathcal {N}} k_{ij} k_{jk} k_{ki}}{\sum _{i,j,k \in \mathcal {N}} k_{ij} k_{jk}}.
\end{eqnarray*}

**Figure 2.**
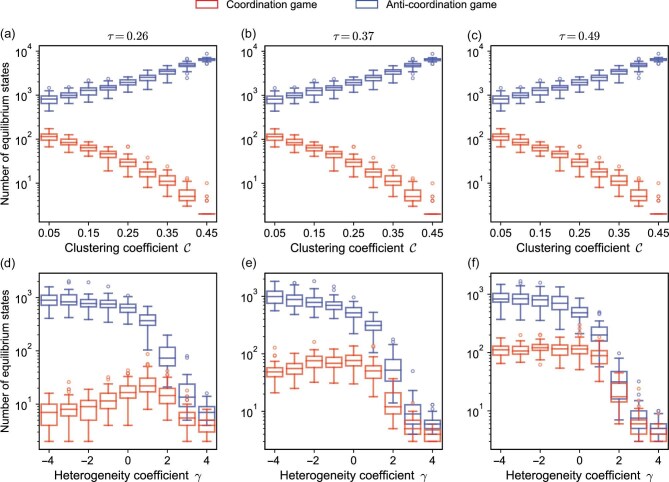
A minor change in network structure can dramatically change the number of equilibrium states. We consider two important network characteristics: closed triads (measured by the clustering coefficient $\mathcal {C}$) and the node degree heterogeneity distribution (measured by the distribution heterogeneity coefficient $\gamma$). We generate networks with varying clustering coefficients by rewiring edges in a degree-preserving manner, starting with regular ring lattice networks [50]. Networks with different heterogeneity coefficients are generated based on the connection kernel and the preferential attachment mechanism [38,51]. The number of equilibrium states is presented for a broad range of clustering coefficients $\mathcal {C}$ (a–c) and heterogeneity coefficients $\gamma$ (d–f), for three thresholds, $\tau = 0.26, 0.37, 0.49$, and two types of games, coordination and anti-coordination. For each kind of network, we sample 50 networks and present the median, quartiles and outliers. Modifications to network structures significantly change the number of equilibrium states. Parameters: $N=30$ and average degree $\bar{k}=4$.

Here, we perform an exhaustive search of the entire state space to identify and count all possible equilibrium states. In Fig. 2a–c, we show the number of equilibrium states as a function of the clustering coefficient. As the clustering coefficient increases, there is a decrease in the number of equilibrium states in coordination games, while this number increases in anti-coordination games. For coordination games in networks with high clustering coefficients, if interconnected nodes choose the same strategy then it becomes highly probable for their common neighbors to adopt the strategy, eventually resulting in the strategy being employed by the vast majority or even all nodes. Therefore, the number of equilibrium states in networks with high clustering coefficients is typically lower than that in networks with low clustering coefficients. Consequently, the number of equilibrium states in coordination games is low because the entire network essentially forms a large cluster. In contrast, for anti-coordination games in networks with high clustering coefficients, individuals choose strategies opposite to most of their neighbors. Therefore, if the strategy proportions within the clusters remain at the same level, more individuals can choose between two strategies, culminating in a greater number of equilibrium states (see Fig. S2 for an example of the effect of clustering coefficients under different games). Thus, clusters promote the spread of strategies in coordination games, but act as a barrier in anti-coordination games. In other words, clusters are usually taken over by one strategy in coordination games, while they always include two opposing strategies in anti-coordination games.

We also investigate how degree heterogeneity, measured by the heterogeneity coefficient $\gamma$, affects the number of equilibrium states. This coefficient parametrizes a connection kernel for preferential attachment networks, with a new node being connected to a degree-*k* node with probability proportional to $k^{\gamma }$ [51]. Heterogeneity in the degree distribution grows with $\gamma$. Notably, at $\gamma =1$, networks exhibit scale-free characteristics. Meanwhile, networks with $\gamma <0$ are akin to random networks, and those with $\gamma >1$ are more closely related to star networks (see Fig. S3 for the degree distribution of these networks). Figure 2d–f illustrate that the number of equilibrium states decreases with more degree heterogeneity in both coordination and anti-coordination games. In contrast to homogeneous networks, heterogeneous networks typically have hubs whose degree greatly exceeds the average. In these networks, individuals tend to adopt the same (respectively opposite) strategy as that of the majority of hubs in coordination (respectively anti-coordination) games. At the same time, as degree heterogeneity intensifies, the number of hubs decreases due to the unchanged total edge count, which means that the number of equilibrium states is determined by the number of hubs at high $\gamma$ values. Therefore, we observe that in heterogeneous networks, the number of equilibrium states is markedly low in both coordination and anti-coordination games compared with homogeneous networks, with the count of equilibrium states being very close under both types of games in such networks (see Fig. S4 for an example). These results highlight the dominance of hubs in both coordination and anti-coordination games.

### A simple network measure capturing evolutionary outcomes

**Figure 3.**
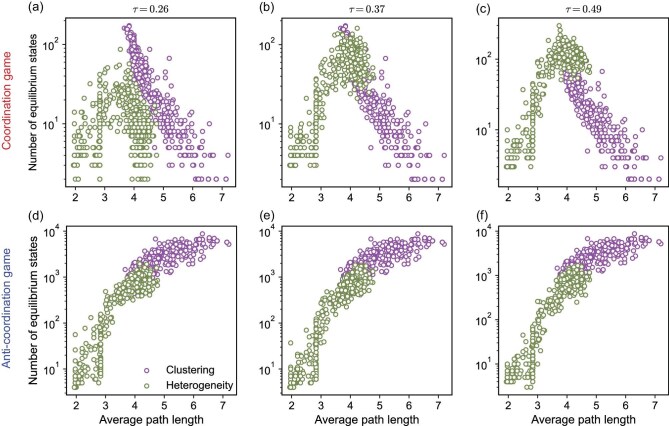
The number of equilibrium states as a function of average path length. Panels (a) and (d) correspond to a $\tau$ value of 0.26, (b and e) to a $\tau$ value of 0.37, and (c and f) to a $\tau$ value of 0.49. We investigate networks with different structures, including those with variations in clustering coefficients (corresponding to Fig. 2a–c) and in degree distribution heterogeneity (corresponding to Fig. 2d–f). Every dot is the number of equilibrium states of a network. For a given type of game, the average path length describes the number of equilibrium states: a moderate average path length leads to a higher number of equilibrium states in coordination games (a–c), whereas in anti-coordination games, a greater average path length results in a higher number of equilibrium states (d–f). Parameter values: network size $N=30$ and average degree $\bar{k}=4$.

Both the clustering coefficient and degree distribution are fundamental properties of complex networks, and can have strong impacts on evolutionary dynamics. An important quantity in the graphs shown in Fig. 2 is the average path length, which represents the mean number of edges that nodes in the network have to pass through to reach the most distant node. By reorganizing the data presented in Fig. 2 and modifying the horizontal axis to represent the average path length, we obtain Fig. 3. Here, the clustering coefficient is positively correlated with the average path length, and the heterogeneity coefficient is negatively correlated with it (see Fig. S24 for details). Figure 3 shows that, as the average path length increases, the number of equilibrium states increases, peaks and then decreases in coordination games (Fig. 3a–c). On the other hand, anti-coordination games see continual growth in the number of equilibrium states as the average path length of the network grows (Fig. 3d–f).

**Figure 4.**
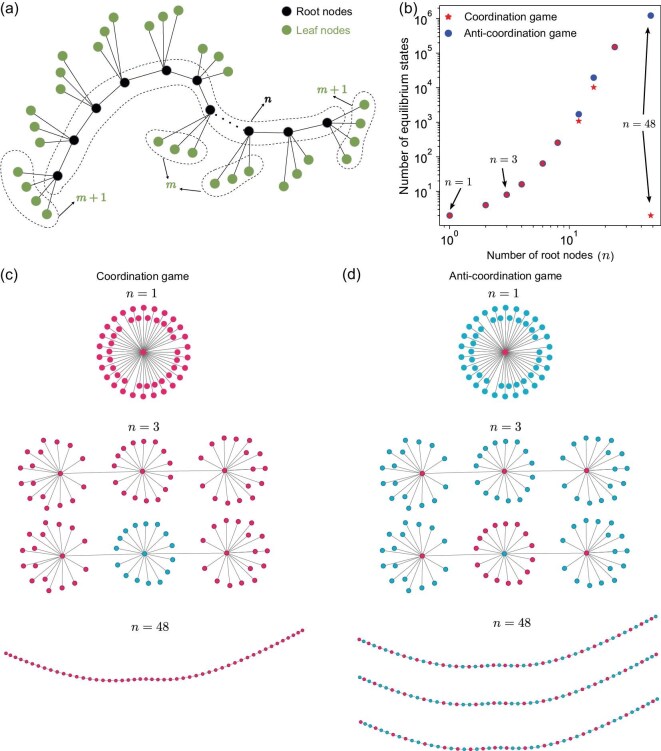
A sequential root-leaf structure. (a) In this idealized structure, *n* root nodes are sequentially connected. Each root node is linked to *m* leaf nodes, with the exception that the terminal root nodes at both ends each connect to $m+1$ leaf nodes. (b) In coordination games, there is an initial increase followed by a decrease in the number of equilibrium states as *n* grows. Conversely, in anti-coordination games, the quantity of equilibrium states grows with *n*. (c) Typical equilibrium states for the sequential root-leaf structure with different root nodes in coordination games. (d) Typical equilibrium states for the sequential root-leaf structure with different root nodes in anti-coordination games. Parameters: network size $N = 50$ and behavioral switching threshold $\tau =0.37$.

To better understand this observation, we can analyze a sequential root-leaf structure, a representative network on which it is possible to more explicitly characterize the number of equilibrium states. This network is parameterized by *n*, the number of hubs, and *m*, the number of leaves per (interior) hub (the terminal nodes have $m+1$ leaves; see Fig. 4a). For a fixed network size, increasing the number of hubs leads to a longer average path length (but requires reducing the number of leaves per hub to maintain constant size). In both coordination and anti-coordination games, we provide an explicit formula for the number of equilibrium states, as a function of the number of root nodes, *n*, leaf nodes, *m*, and the behavioral switching threshold, $\tau$. The basic structure of the analysis is given in Section S2, where the number of equilibrium states is characterized in terms of the eigenvalues of a $4\times 4$ binary matrix.

In a coordination game, when $m\ge 2$ and $2/(m+2)\le \tau \le m/(m+2)$, the number of equilibrium states is $2^{n}$. On the other end of the spectrum, when $m=0$ and $\tau \ne 0.5$, the number of equilibrium states is simply 2, owing to the fact that the network is just a linear structure in which the all-*A* and all-*B* states are the only equilibria (Fig. 4c). For other parameter ranges of *m* and $\tau$, we can characterize the growth of the number of equilibrium states (see the online supplementary material), but the important property is that this structure captures the behavior observed in Fig. 3a–c, in which the number of equilibrium states is maximized at an intermediate average path length (Fig. 4b).

In anti-coordination games, the number of equilibrium states is determined by hubs as well. We note an upward trend in the equilibrium state count with low and increasing values of *n*. But, unlike the coordination game, more equilibrium states exist when *n* is significantly large, corresponding to the cases when $m=0$ and $\tau \ne 1/2$ (Fig. 4b). For example, when $n=48$, $m=0$ and $\tau =0.37$, there are $1\, 221\, 537$ equilibrium states. In fact, hubs vanish in networks with long path lengths, individuals must adopt strategies opposite most of their neighbors (Fig. 4d), thereby introducing more potential equilibria.

### Distribution of equilibrium states

The set of equilibrium states is vast, so it is natural to ask what the differences are among them and which ones are more likely to be reached. In this section, we explore the equilibrium probability distribution of various networks, a classic topic in network dynamics. We begin with a random initial setup in which each individual adopts strategy *A* with probability $1/2$ (and *B* otherwise). Best-response dynamics then unfolds on the network until an equilibrium is reached. This process is repeated many times, and we record the frequency of each equilibrium appearing in the final state as a proxy for the probability of observing various equilibria.

**Figure 5.**
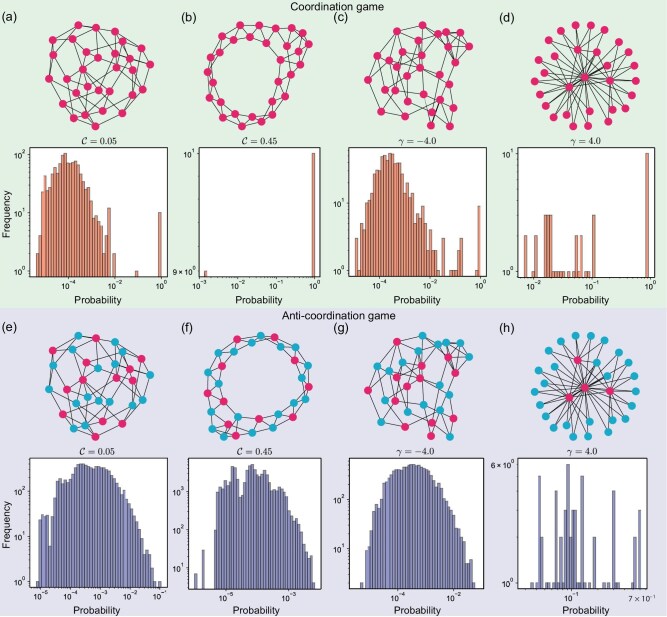
Preferred decision-making patterns in coordination and anti-coordination games. We analyzed the arrival probability of each equilibrium state for both coordination games (a–d) and anti-coordination games (e–f). In each histogram of panels (a–h), the horizontal axis represents the arrival probability, while the vertical axis indicates the number of equilibrium states whose probabilities fall within the corresponding interval. The equilibrium state with the strongest absorption capacity corresponding to each parameter is shown in each panel. Each histogram displays the results from simulations conducted $3\times 10^7$ times on each of 10 distinct networks. Parameter values: network size $N=30$, average degree $\bar{k}=4$ and behavioral switching threshold $\tau = 0.37$.

Figure 5 illustrates the degree distribution when the clustering coefficient is 0.05 and 0.45 and the heterogeneity coefficient is $-4$ and 4. (See Fig. S5 for the distribution under other parameter values.) In the coordination game, there exist equilibrium states with strong absorption capacity in any network. The behavioral switching threshold determines if one strategy is favored over the other: when $\tau <0.5$, strategy *A* is favored in coordination games and disfavored in anti-coordination games. From the data, we find that after half of the individuals in the system adopt the favored strategy, the system tends to evolve into a state where all individuals adopt the same strategy (Fig. S6).

This phenomenon can be explained via the observation that, since each individual adopts strategy *A* with probability equal to 0.5 and the behavioral switching threshold is $\tau <0.5$, the inequality $n_i > \tau k_i$ is likely to hold. Therefore, the probability of reaching the state where all individuals adopt strategy *A* is high. However, in anti-coordination games, only heterogeneous networks have equilibrium states with strong absorption capacity. This occurs because, in heterogeneous networks, hubs exist, and once the strategy of these hubs is determined, the strategies of many of the remaining nodes are determined. Conversely, in homogeneous networks, hubs do not exist. Individuals simply need to be in the minority among their neighbors, indicating that the system lacks a specific driving force to reach a predetermined equilibrium state.

In Fig. 5, we show representative equilibrium states of networks under different parameters (that is, equilibrium states with the highest probability of being reached). We find that the preferred equilibrium states are those in which all individuals adopt the same strategy (the favored strategy) in coordination games (Fig. 5a–d). In contrast, the hubs emerging in heterogeneous networks play a significant role in anti-coordination games. Here, hubs adopt the same strategy and non-hub nodes choose the opposite strategy (Fig. 5h). In homogeneous networks, strategies emerge layer by layer, where ‘layer’ refers to a group of nodes that have few or no direct connections among themselves. Therefore, individuals on nodes of one layer select one strategy, and individuals on an adjacent layer choose the opposing strategy. This phenomenon is particularly evident in networks with high clustering coefficients (Fig. 5f).

### Equilibrium time

In addition to which states the system can reach, an important quantity is the time it takes to reach these states. In this section, we consider the equilibrium time, which is measured by the total number of strategy changes in the system prior to reaching an equilibrium state.

**Figure 6.**
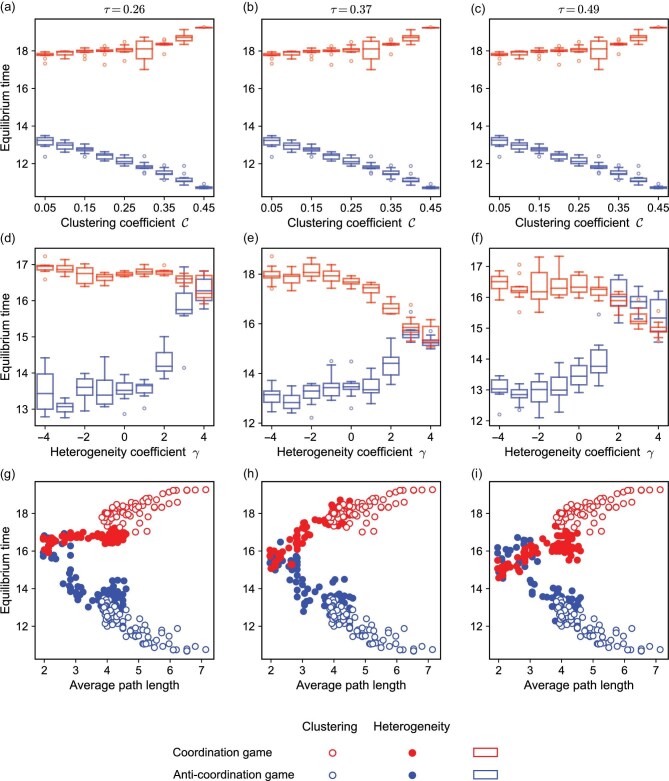
Equilibrium time as a function of the clustering coefficient, heterogeneity and the average path length. The average equilibrium time of different networks is shown for coordination games and for anti-coordination games. Each box in panels (a–f) represents the average equilibrium time for 50 networks, showing the median, quartiles and outliers. Open and filled circles in (g–i) correspond to the data in (a–c) and (d–f), respectively. Panels (a), (d) and (g) correspond to a $\tau$ value of 0.26, (b), (e) and (h) to a $\tau$ value of 0.37, and (c), (f) and (i) to a $\tau$ value of 0.49. The average equilibrium time increases (decreases) in coordination (anti-coordination) games as the clustering coefficient increases, while it decreases (increases) in coordination (anti-coordination) games as the heterogeneity coefficient increases. (g–i) The equilibrium time increases in coordination games and decreases in anti-coordination games as the average path length increases. The simulation is repeated $3\times 10^7$ times for each network. Parameter values: network size $N=30$ and average degree $\bar{k}=4$.

Figure 6a–c demonstrate that in coordination games, the average time to reach an equilibrium increases as the clustering coefficient increases. We know already that strategies diffuse in a clustered pattern in coordination games. A high clustering coefficient enables the favored strategy to dominate the entire network, while one strategy spreading throughout the network requires the adjustment of strategies in a larger number of individuals, thereby increasing the average equilibrium time. On the other hand, increasing degree heterogeneity leads to a reduction in the average equilibrium time in coordination games (Fig. 6d–f). In homogeneous networks, these games have equilibrium states with strong absorption capacity, and such equilibria can be reached from very different initial states, increasing the equilibrium time. In heterogeneous networks, hubs dominate the system, which leads to faster equilibration times.

Conversely, we observe coexistence of strategies *A* and *B* in anti-coordination games. Thus, to keep the strategy proportions stable among the clusters, only relatively few nodes need to switch strategies, leading to a decrease in the average time to achieve equilibrium in networks with high clustering coefficients (Fig. 6a–c). We also find that greater degree heterogeneity results in a longer equilibrium time in anti-coordination games (Fig. 6d–f). Other nodes must choose the opposite strategy to the hubs, resulting in more strategy changes.

We can derive intuition about these outcomes by examining the number of equilibrium states. More equilibrium states lead (in principle) to more evolutionary outcomes. Consider two extreme cases: all states are equilibria and only one state is an equilibrium. If all the states are equilibria then no matter what the initial state is, the system reaches the equilibrium state directly without any strategy changes. Conversely, if there is only one equilibrium state and the initial states are uniformly distributed, many more strategy changes are required (on average). Therefore, at least informally, systems with fewer equilibrium states need more strategy changes to reach an equilibrium state. In coordination games, networks with high clustering coefficients have fewer equilibrium states than those with lower clustering coefficients (Fig. 2a–c), thus reaching equilibrium slower. A similar logic applies to anti-coordination games.

Once again, the effects of network structure on equilibrium time can be understood via the basic network metric of average path length. Figure 6g–i depict the variation in average equilibrium time as the average path length changes. We analyze all the states of the star (Figs S7 and S8) and the chain network (Figs S9 and S11) of size six. We find that the average equilibrium time grows (shrinks) as the average path length increases under coordination (anti-coordination) games.

Networks with short path lengths tend to have hubs, and the strategies of all other nodes are restricted by these hubs. Thus, networked systems with short path lengths generally converge rapidly to equilibrium states in both coordination and anti-coordination games. The star graph of size six illustrates this conclusion (Figs S7 and S8 for examples). In contrast, hub nodes tend not to exist in networks with long path lengths, and some nodes switch strategies twice before reaching equilibrium. For instance, consider the linear segment $ABABA$. Before eventually reaching the all-*A* state, it may first transition through $ABBBA$ and then become $AAAAA$; the middle *A* individual switches its strategy twice. In the chain network of size six, the equilibrium states where all individuals choose strategy *A* have strong absorption capacity in coordination games (Fig. S9). Before all individuals adopt strategy *A*, there must be two interconnected nodes (individuals) employing strategy *A*, and the *A* node (individual) without an *A* neighbor might first switch to strategy *B* (Fig. S10 for an example).

In anti-coordination games, however, due to the repulsion between strategies of the same type, individuals adjust their strategies to become the minority among those of their connections. For instance, in the linear segment $AAAAA$, after two of the individuals switch to strategy *B*, yielding $ABABA$, all individuals tend to keep their strategy unchanged. In this case, strategy *B* spreads through the network in a leapfrog manner, meaning that it bypasses immediate neighbors and instead spreads to the neighbors of neighbors. Therefore, in networks with long average path lengths, only a few individuals need to adjust their strategies to ensure that all connected individuals’ strategies are the best response to their neighbors, expediting the transition to an equilibrium state (see Fig. S12 for an example).

### Robustness

The robustness of dynamical systems is a topic that has long been a concern in many fields [55–57]. Here, we explore the robustness of different networks by starting a network in an equilibrium state, perturbing it and then measuring how many strategy changes are required to return to an equilibrium. The initial equilibrium state is selected based on the distribution of equilibrium states, implying that an equilibrium state reached with greater frequency is more likely to be chosen as the initial state. For a given initial state, we perturb the network by adding a single, randomly chosen edge. If $s_{i}(0)$ and $s_{i}^{\ast }$ denote the state of node *i* prior to the perturbation and after equilibrium has been restored following the perturbation, respectively, then we measure robustness of the network via the average number of strategy changes,


(6) \begin{eqnarray*}
\Delta _{s} = \sum _{i \in \mathcal {N}} |s_{i}^{\ast } - s_{i}(0)| .
\end{eqnarray*}

**Figure 7.**
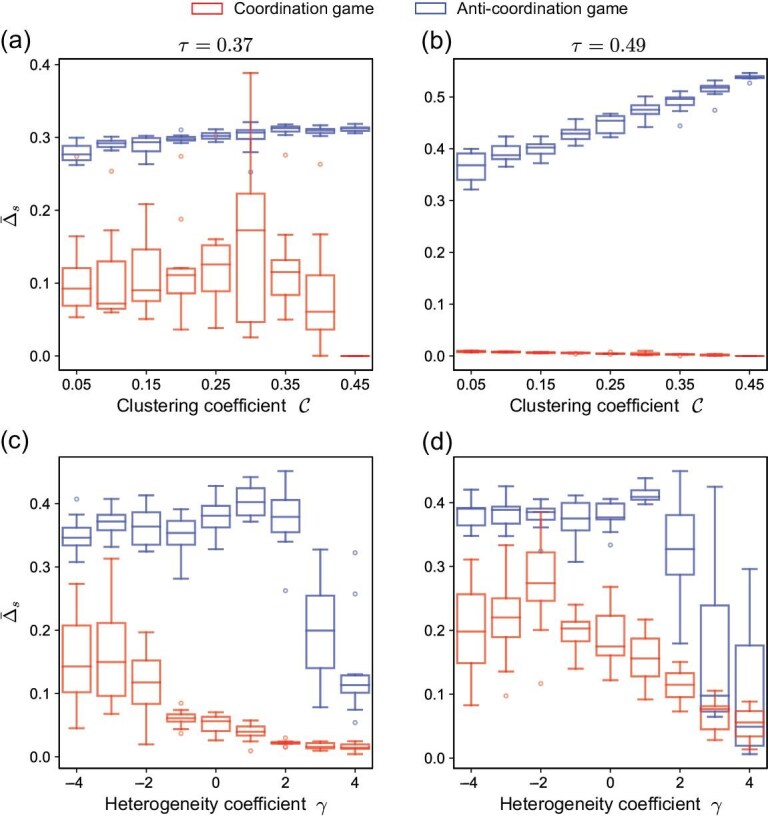
Coordination games are more robust than anti-coordination games. We assess the robustness of the system by randomly adding a new edge to the equilibrium state and measuring the resulting distance (i.e. the number of strategy changes required to reach the final equilibrium state from the initial equilibrium state). The red (blue) boxes represent the average number of strategy changes in coordination (anti-coordination) games, and each of them is the average over 20 networks. (a and b) The average number of strategy switches varies with changes in clustering coefficients. (c and d) The average number of strategy switches varies with changes in degree heterogeneity. We used a network size of $N=30$ and an average degree of $\bar{k}=4$.

In most cases, networks with coordination games exhibit greater robustness than those with anti-coordination games (Fig. 7). For networks with coordination games, in the equilibrium state with the strongest absorption capacity, all individuals adopt the same strategy (Fig. 5), thus adding new edges does not have an effect according to Equation (3). For networks with anti-coordination games, there exist no equilibrium states with strong absorption capacity except in heterogeneous networks. Thus, in most cases, adding edges causes more individuals to change strategies in anti-coordination games, resulting in larger values of $\Delta _{s}$.

However, in some equilibrium states for coordination games in which two strategies coexist, adding a new edge can drive the system to a monomorphic state (see Fig. S13 for an example). Thus, adding new edges to networks in coordination games may result in several individuals switching strategies when their neighbors with strategy *A* increase and the value of $\lfloor \tau k \rfloor$ remains constant. This is why there are particularly sensitive networks when the clustering coefficient equals 0.30 in Fig. 7a. Meanwhile, in heterogeneous networks, fewer individuals tend to switch their strategy than they do in homogeneous networks. Hubs, which are often present in heterogeneous networks, tend to maintain their current strategy under small perturbations, and the strategy changes among other nodes cannot influence the hubs, yielding greater robustness.

Our work focuses mainly on populations in which all individuals have the same behavioral switching threshold. In the online supplementary material, we consider a complete bipartite graph with nodes divided into two disjoint subsets: one ‘left’ set containing $n_l$ nodes and threshold $\tau _l$, and the other ‘right’ set containing $n_r$ nodes and threshold $\tau _r$ (see Section S4 for details). When $\tau _l > n_r/ (n_r+1)$ and $\tau _r <1/n_l$, and initially the left set uses *B* and the right set uses *A*, adding an edge to connect two nodes in the left subset leads to an inversion in which the left set uses *A* and the right set uses *B*. Thus, even a small perturbation can result in dramatic changes in systems with non-uniform thresholds. We also investigate examples of adding more than one edge to the system. The results are similar to those shown here (Fig. S14).

### Real-world networks

**Figure 8.**
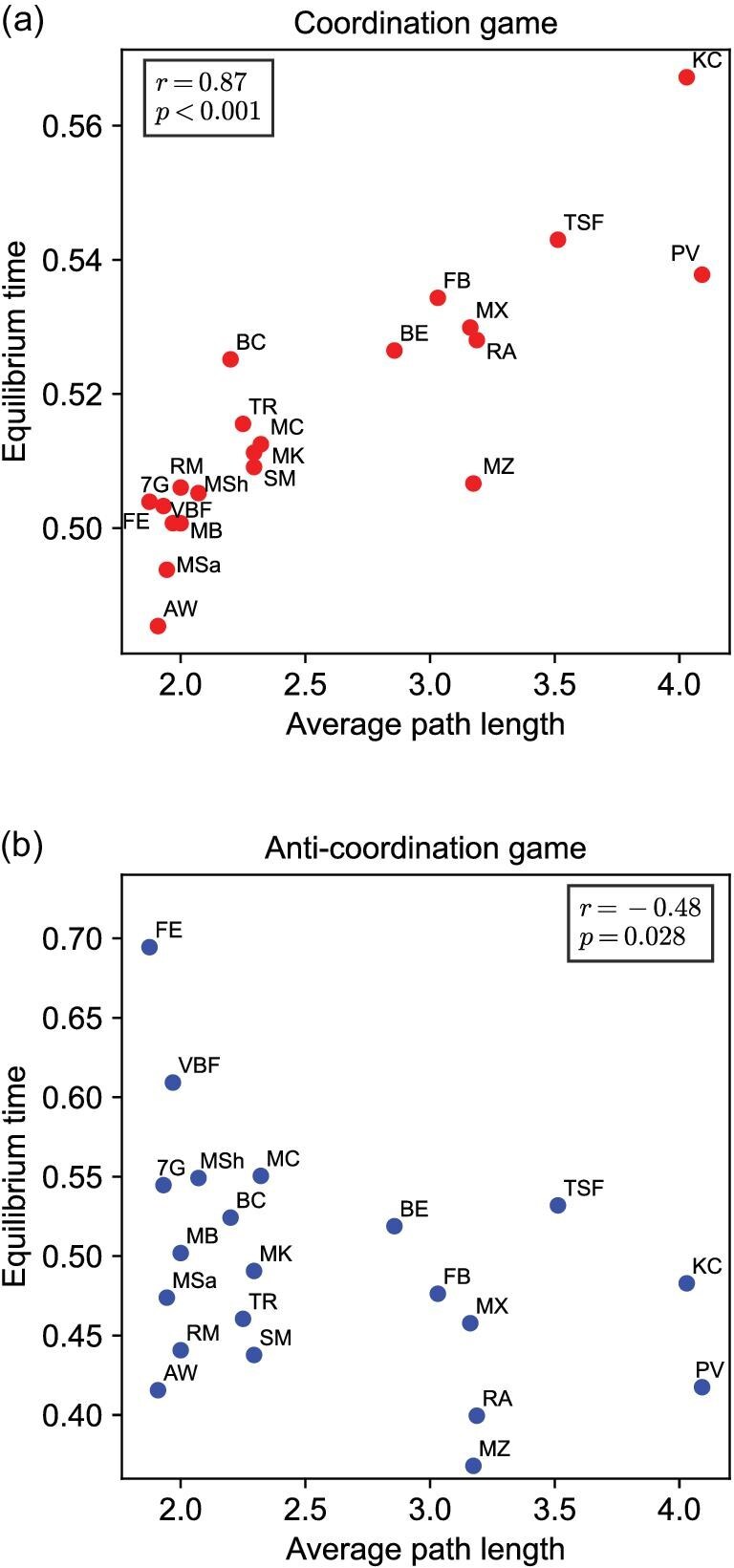
Average path length determines the equilibrium time in empirical networks. The equilibrium time of 20 real-world networks for both coordination games (a) and anti-coordination games (b) is shown as a function of the average path length. The equilibrium time of each network is normalized by *N*. The equilibrium time increases in coordination games and decreases in anti-coordination games as the average path length of empirical networks increases.

Here we analyze the system dynamics across 20 empirical networks (see Table S4 for details) that span a wide range of real-world contexts, including physical and online social relationships (e.g. school friendships and Facebook ties), workplace and organizational structures (e.g. CEO clubs and employee interactions), small-scale communities (e.g. villages and tribal societies) and animal social systems (e.g. bison and sheep). These networks vary substantially in size (from $N=16$ to $N=39$) and average degree (from $\bar{k}=3.55$ to $\bar{k}=22.25$), reflecting the structural diversity of natural and social systems. In each empirical network, we simulate strategy updates from random initial conditions and record the average number of time steps required to reach equilibrium, normalized by the network size *N*. Figure 8 demonstrates how average path length systematically shapes equilibrium time in empirical networks. We find that equilibrium time increases with average path length in coordination games (Fig. 8a), whereas it decreases in anti-coordination games (Fig. 8b). We calculate the Pearson correlation coefficient *r*, which measures the strength and direction of the linear relationship between two variables. Specifically, $r>0$ means that larger values of one variable tend to be associated with larger values of the other, $r<0$ means that larger values of one tend to be associated with smaller values of the other, and $|r|$ closer to 1 indicates a stronger relationship. To check whether the observed correlation could simply be due to random chance rather than a real link between the variables, we use a permutation test to calculate the *P*-value to assess statistical significance. The resulting trends reveal a significant positive correlation between average path length and equilibrium time in coordination games ($r = 0.87$, $P < 0.001$), and a significant negative correlation in anti-coordination games ($r = -0.48$, $P = 0.028$), corroborating the patterns previously identified in synthetic network models such as random-regular and scale-free networks. Therefore, such patterns are not artifacts of any individual network, but reflect a robust principle generalizing across diverse empirical systems. These findings highlight the predictive power of average path length and support its theoretical importance in shaping system dynamics in strategic interactions, both in controlled models and in complex, real-world environments.

## DISCUSSION

In this paper, we analyze the interplay between network structure and best-response dynamics, emphasizing the behavioral patterns arising in both coordination and anti-coordination games. We find that coordination games typically exhibit fewer equilibrium states, greater equilibration times and more robustness than anti-coordination games. The comparison between coordination games and anti-coordination games on a common network reveals fundamental differences between the two behavioral patterns, even though the underlying logic of decision-making at an individual level is to maximize payoff. In addition, we systematically study the influence of spatial structure, investigating how closed triads and hubs (two important components in complex networks) affect system dynamics. We find a simple measure, the average path length in the network, that serves as a good indicator of the resulting dynamics.

Our analysis of network properties, including the clustering coefficient, heterogeneity coefficient and average path length, applies for general complex networks (e.g. Erdős-Rényi, scale-free, small-world and empirical networks). However, in highly structured special cases, exceptions to our findings may arise. In the complete bipartite graph, for example, there are typically only two equilibrium states in both coordination and anti-coordination games (see Section S4). This finding lies in contrast to our previous finding that in networks with low clustering coefficients, there are more equilibrium states in coordination games. The number of equilibrium states can fluctuate dramatically when the behavioral switching threshold changes, especially if its product with the degree of the highest frequency in the network is an integer (see Figs S15 and S16 for examples). An integer product makes it possible for equality to hold in Equations (3) and (4), and such individuals are free to adopt either strategy *A* or *B* in an equilibrium, resulting in a greater quantity of equilibrium states overall.

Unlike reinforcement learning, our model is grounded in introspection-based dynamics, where individuals choose strategies based on neighbors’ behaviors in the immediately preceding round and aim only to improve the current payoff. Compared to link-prediction studies, which aim to infer missing edges from existing network structures and may treat circle-like or tree-like topologies as prediction targets [58,59], our study adopts a different perspective. Here, the network is used as a fixed experimental condition to examine how structural properties, particularly the average path length, influence the evolutionary dynamics and equilibrium outcomes of coordination and anti-coordination systems. Unlike prior data-driven simulations of multi-dimensional opinion dissemination for specific events [60], our study provides a rigorous theoretical framework for coordination and anti-coordination dynamics on graphs, and finds that the average path length can serve as a general structural metric, which holds across a variety of networked systems.

Imitation, along with coordination and anti-coordination, are all significant heuristic decision-making patterns. Imitation has received considerable attention due to the potential of imitation systems to complete complex tasks under simple rules [8,61–63]. However, the advantages of coordination and anti-coordination games in this regard are more substantial than those of imitation. In imitation systems, decision-makers need not discern only the behavioral information of the individuals they interact with, but also the payoffs resulting from these behaviors. On the other hand, in coordinating and anti-coordinating systems, decision-makers can make decisions based only on the observed behaviors of others. Compared with aspiration dynamics [64], which also relies on local information, our model uses observed behaviors of others while aspiration dynamics uses internal benchmarks. They also differ in structural sensitivity: aspiration dynamics is robust to network variations, whereas our model exhibits a systematic dependence on network topology, governed by the average path length. The distinction between fixation time in evolutionary game theory [65–67] and equilibrium time discussed here lies in their respective focuses: fixation time quantifies the time for a single mutant trait to become fixed in the system, whereas our notion of equilibrium time captures the time required to reach an equilibrium state from a randomly initialized state. In other words, fixation time is a specific measure of a particular property, while equilibrium time assesses the system’s overall relaxation time.

Best-response dynamics, an introspective learning pattern, is quite different from social learning patterns, such as death-birth or imitation rules that have long been studied in the field of evolutionary dynamics. Some researchers study this topic under probabilistic decision-making in which individuals choose the strategy yielding the highest payoff or imitate the most successful neighbors according to a given probability distribution, and they reveal the distinction between the two learning patterns [35]. Here, we analyze this problem under deterministic decision-making. The idea is that, with sufficient rationality, decision-makers can find the optimal solution as the best response to the environment [68,69]. Best-response dynamics may be viewed as an extreme version of logit-response dynamics [70], which is parameterized by a rationality term. As this rationality level approaches zero, an individual adopts actions uniformly at random. As it approaches infinity, an individual adopts actions based on payoff maximization, yielding best-response dynamics in the limit. In between, rationality is bounded [71], and although better-performing actions are favored, there is the potential for suboptimal play. Humans do not always behave perfectly rationally, and, as a result, it could be the case that some states that appear to be equilibria are technically not equilibria, but still there should be some exponential escape time from a neighborhood of these states, making them quasi-equilibria.

Looking ahead, it is crucial to develop and refine computational methods aimed at calculating (or approximating) the equilibrium distribution and times, as these aspects have implications for many real-life scenarios. For example, voting processes can be modeled as coordinating systems [72,73], and understanding both the transient dynamics and equilibria can inform strategies for targeted information delivery. In non-human systems such as power grids, interpreting strategies in the network in terms of resource distribution allows one to study efficient allocation of resources (this would typically be an anti-coordinating system). Of course, in these systems and others, networks can be highly heterogeneous and individuals may not always have the same behavioral switching thresholds as we have assumed here. Stochasticity and multi-player interactions are common in collective systems. For instance, stochasticity may result from random decision errors, while multi-player interactions can arise when multiple individuals contribute to a shared resource. Recent studies have shown that such factors can fundamentally alter the underlying dynamics [74–77], leading to qualitatively different equilibrium structures and evolutionary outcomes. In the context of networked systems with best-response decision rules, exploring how stochasticity and multi-player interactions jointly shape system dynamics represents a challenging yet highly meaningful direction for future research.

## Supplementary Material

nwaf403_Supplemental_File
