# Supplementary material for: Behavioral alignment in social networks
Source: Natl Sci Rev. 2025 Sep 19;12(11):nwaf403. doi: 10.1093/nsr/nwaf403 (PMC12553140; doi:10.1093/nsr/nwaf403)
Supplement: nwaf403_Supplemental_File [file nwaf403_supplemental_file.pdf]

Supplementary Information for

Behavioral alignment in social networks

1 Model

The system is described by a network consisting of N nodes, which are denoted by $\mathscr{N} = \{1,2,\ldots,N\}$. The edges are tracked by an adjacency matrix $\{k_{ij}\}_{i,j\in\mathscr{N}} \in \{0,1\}$, where $k_{ij} = k_{ji}$ because the interaction is symmetric. Here $k_{ij} = 1$ means individual i and j interact with each other, while $k_{ij} = 0$ indicates that they do not. If $i = j$, then $k_{ij} = 0$ (that is, there are no-self loops). The total degree of node i is $k_i := \sum_{j\in\mathscr{N}} k_{ij}$.

Individuals choose their strategy from the set $\mathscr{S} = \{A,B\}$. When individual i selects strategy A and one of its neighbors uses strategy A (or B), it receives a payoff of a_i (or b_i). Similarly, if individual i chooses strategy B and one of its neighbors adopts strategy A (or B), it obtains a payoff of c_i (or d_i). Let $s_i(t)$ denote individual i's strategy at time t, where $s_i(t) = 1$ represents strategy A and $s_i(t) = 0$ represents strategy B. The total payoff to i is then

$$u_i(t) = \sum_{j\in\mathscr{N}} k_{ij}\left(s_i(t)\left(a_i s_j(t) + b_i\left(1 - s_j(t)\right)\right) + \left(1 - s_i(t)\right)\left(c_i s_j(t) + d_i\left(1 - s_j(t)\right)\right)\right). \quad (1)$$

At each time step, a random individual is selected to update its strategy. Under best-response dynamics, the chosen individual tries to maximize $u_i(t)$, adopting the strategy that leads to a higher payoff.

The number of i's neighboring individuals adopting strategy A (B) at time t is denoted by $n_i(t)$ ($k_i - n_i(t)$). Define the behavioral switching threshold of individual i as $\tau_i = (d_i - b_i)/(a_i + d_i - b_i - c_i)$. By comparing the payoff generated by strategy A and B, we get the best-response dynamics of individual i. In a coordination game, which satisfies $a_i > c_i$ and $d_i > b_i$ we find that

$$s_i(t+1) = \begin{cases} 1 & n_i(t) > \tau_i k_i, \\ s_i(t) & n_i(t) = \tau_i k_i, \\ 0 & n_i(t) < \tau_i k_i. \end{cases} \quad (2)$$

In anti-coordination games, $c_i > a_i$ and $b_i > d_i$, which gives

$$s_i(t+1) = \begin{cases} 1 & n_i(t) < \tau_i k_i, \\ s_i(t) & n_i(t) = \tau_i k_i, \\ 0 & n_i(t) > \tau_i k_i. \end{cases} \tag{3}$$

2 Sequential root-leaf structure

2.1 Analysis of the number of equilibrium states

As shown in Fig. 4**a** of the main text, in the sequential root-leaf structure the root nodes are connected sequentially, and each root node is connected to m leaf nodes ($m+1$ for the terminal root nodes). For a fixed network size, increasing the number of hubs leads to a longer average path length (but requires a decrease in the number of leaves per hub to maintain constant size). The strategy of node $i \in \mathcal{N}$ at time t is denoted by $s_i(t) \in \{0,1\}$, where $s_i(t) = 1$ and $s_i(t) = 0$ correspond to strategies A and B, respectively. Here, we consider the scenario in which all individuals have the same behavioral switching threshold, meaning $\tau_i = \tau_j = \tau$ for all $i, j \in \mathcal{N}$. Denote the strategy of node i in the equilibrium state as s_i^* and the number of equilibrium states as $f(n,m,\tau)$, which is a function of the number of root nodes, n; the number of leaf nodes, m; and the behavioral switching threshold, τ.

In an equilibrium state, a leaf node's strategy must either match that of the corresponding root (in a coordination game) or be of the opposite type (in an anti-coordination game). Therefore, we can define a reduced, elementary (and totalistic) graph automaton with the same number of fixed points as the full system, which can then be used to study the number of equilibrium states. In what follows, we let a_n, b_n, c_n, and d_n denote the number of equilibrium states in the relevant elementary automaton of length n, when the leftmost states are AA, AB, BA, and BB, respectively. For $X,Y,Z \in \{A,B\}$, let $S\left(\boxed{X}Y\right)$ and $S\left(X\boxed{Y}Z\right)$ be indicators for whether X in the endpoint XY and Y in the interior triple XYZ are stable, respectively. (Note that, since the automaton is totalistic, we have $S\left(X\boxed{Y}Z\right) = S\left(Z\boxed{Y}X\right)$ for all $X,Y,Z \in \{A,B\}$.)

For $n > 2$, we have

$$a_n = S\left(A\boxed{A}A\right) a_{n-1} + S\left(A\boxed{A}B\right) b_{n-1}; \tag{4a}$$

$$b_n = S\left(A\boxed{B}A\right) c_{n-1} + S\left(A\boxed{B}B\right) d_{n-1}; \tag{4b}$$

$$c_n = S\left(A\boxed{A}B\right) a_{n-1} + S\left(B\boxed{A}B\right) b_{n-1}; \tag{4c}$$

$$d_n = S\left(A\boxed{B}B\right) c_{n-1} + S\left(B\boxed{B}B\right) d_{n-1}. \tag{4d}$$

The boundary conditions are

$$a_2 = S\left(\boxed{A}A\right); \tag{5a}$$

$$b_2 = S\left(\boxed{A}B\right); \tag{5b}$$

$$c_2 = S\left(\boxed{B}A\right); \tag{5c}$$

$$d_2 = S\left(\boxed{B}B\right). \tag{5d}$$

More compactly, we can write

$$\begin{aligned}
\begin{pmatrix} a_n \\ b_n \\ c_n \\ d_n \end{pmatrix} &= \begin{pmatrix} S\left(A\boxed{A}A\right) & S\left(A\boxed{A}B\right) & 0 & 0 \\ 0 & 0 & S\left(A\boxed{B}A\right) & S\left(A\boxed{B}B\right) \\ S\left(A\boxed{A}B\right) & S\left(B\boxed{A}B\right) & 0 & 0 \\ 0 & 0 & S\left(A\boxed{B}B\right) & S\left(B\boxed{B}B\right) \end{pmatrix} \begin{pmatrix} a_{n-1} \\ b_{n-1} \\ c_{n-1} \\ d_{n-1} \end{pmatrix} \\
&= \begin{pmatrix} S\left(A\boxed{A}A\right) & S\left(A\boxed{A}B\right) & 0 & 0 \\ 0 & 0 & S\left(A\boxed{B}A\right) & S\left(A\boxed{B}B\right) \\ S\left(A\boxed{A}B\right) & S\left(B\boxed{A}B\right) & 0 & 0 \\ 0 & 0 & S\left(A\boxed{B}B\right) & S\left(B\boxed{B}B\right) \end{pmatrix}^{n-2} \begin{pmatrix} a_2 \\ b_2 \\ c_2 \\ d_2 \end{pmatrix}.
\end{aligned} \tag{6}$$

(For later use, we denote by M the matrix in Eq. 6.) In total, the number of fixed points is

$$f(m,n,\tau) = S\left(\boxed{A}A\right)a_n + S\left(\boxed{A}B\right)b_n + S\left(\boxed{B}A\right)c_n + S\left(\boxed{B}B\right)d_n = z^{\mathsf{T}}M^{n-2}z, \tag{7}$$

where $z^{\mathsf{T}} = \begin{pmatrix} a_2 & b_2 & c_2 & d_2 \end{pmatrix}$. For the values that M can take (see below), it must be true that either $M^3 = M^2$ or M is diagonalizable. In the former case, $M^n = M^2$ for all $n \geqslant 2$. In the latter case, we let $v_1, v_2, v_3, v_4 \in \mathbb{C}^4$ be an eigenbasis for M, corresponding to eigenvalues $\lambda_1, \lambda_2, \lambda_3, \lambda_4 \in \mathbb{C}$, respectively. If D is the diagonal matrix of eigenvalues and V is the matrix whose columns are the eigenvectors, then

$$f(m,n,\tau) = z^{\mathsf{T}}M^{n-2}z = z^{\mathsf{T}}VD^{n-2}V^{-1}z = \sum_{i=1}^{4} \lambda_i^{n-2}\left(z^{\mathsf{T}}V\right)_i\left(V^{-1}z\right)_i. \tag{8}$$

This expression can be evaluated simply by computing z and the eigenvalues and eigenvectors of M, and the result will be an integer even when some of these constituent terms are complex.

All the same, it can be useful to write this expression slightly differently when some of these terms have non-zero imaginary parts. For the matrices M appearing below, either zero or two of the eigenvalues are strictly complex. In the latter case, we let λ_1 and λ_2 be complex

conjugates (because M is real), while λ_3 and λ_4 are real. Let $\lambda_1 = \lambda_1^r + i\lambda_1^i$ with corresponding eigenvector $v_1 = v_1^r + iv_1^i$. Since

$$Mv_1 = Mv_1^r + iMv_1^i = \lambda_1^r v_1^r - \lambda_1^i v_1^i + i\left(\lambda_1^i v_1^r + \lambda_1^r v_1^i\right), \tag{9}$$

we have $Mv_1^r = \lambda_1^r v_1^r - \lambda_1^i v_1^i$ and $Mv_1^i = \lambda_1^i v_1^r + \lambda_1^r v_1^i$. Thus,

$$M \underbrace{\begin{pmatrix} v_1^r & -v_1^i & v_3 & v_4 \end{pmatrix}}_{\widetilde{V}} = \underbrace{\begin{pmatrix} v_1^r & -v_1^i & v_3 & v_4 \end{pmatrix}}_{\widetilde{V}} \begin{pmatrix} \lambda_1^r & -\lambda_1^i & 0 & 0 \\ \lambda_1^i & \lambda_1^r & 0 & 0 \\ 0 & 0 & \lambda_3 & 0 \\ 0 & 0 & 0 & \lambda_4 \end{pmatrix}. \tag{10}$$

If θ_1 is the phase of λ_1 and $|\lambda_1| = \sqrt{\left(\lambda_1^r\right)^2 + \left(\lambda_1^i\right)^2}$ is its modulus, then we obtain

$$f\left(m,n,\tau\right) = z^{\mathsf{T}} \widetilde{V} \begin{pmatrix} |\lambda_1|^{n-2} \cos\left(n-2\right)\theta & -|\lambda_1|^{n-2} \sin\left(n-2\right)\theta & 0 & 0 \\ |\lambda_1|^{n-2} \sin\left(n-2\right)\theta & |\lambda_1|^{n-2} \cos\left(n-2\right)\theta & 0 & 0 \\ 0 & 0 & \lambda_3^{n-2} & 0 \\ 0 & 0 & 0 & \lambda_4^{n-2} \end{pmatrix} \widetilde{V}^{-1} z, \tag{11}$$

which expresses f in terms of only real constituent terms.

2.1.1 Coordination game

Consider the elementary automaton defined as follows. For interior root nodes,

$$\widetilde{s}_i\left(t+1\right) = \begin{cases} 0 & m + \widetilde{s}_{i-1}\left(t\right) + \widetilde{s}_{i+1}\left(t\right) < \left(m+2\right)\tau, \\ 1 & \widetilde{s}_{i-1}\left(t\right) + \widetilde{s}_{i+1}\left(t\right) > \left(m+2\right)\tau, \\ \widetilde{s}_i\left(t\right) & \text{otherwise.} \end{cases} \tag{12}$$

For the terminal root nodes,

$$\widetilde{s}_i\left(t+1\right) = \begin{cases} 0 & m + 1 + \widetilde{s}_{i,\text{neighbor}}\left(t\right) < \left(m+2\right)\tau, \\ 1 & \widetilde{s}_{i,\text{neighbor}}\left(t\right) > \left(m+2\right)\tau, \\ \widetilde{s}_i\left(t\right) & \text{otherwise.} \end{cases} \tag{13}$$

The fixed points of this automaton are in one-to-one correspondence with the equilibrium points of the sequential root-leaf structure.

Component	Condition
$\boxed{A}A$	$\tau \leq 1$
$\boxed{A}B$	$\tau \leq \frac{m+1}{m+2}$
$\boxed{B}A$	$\tau \geq \frac{1}{m+2}$
$\boxed{B}B$	$\tau \geq 0$
$A\boxed{A}A$	$\tau \leq 1$
$A\boxed{A}B$	$\tau \leq \frac{m+1}{m+2}$
$B\boxed{A}B$	$\tau \leq \frac{m}{m+2}$
$A\boxed{B}A$	$\tau \geq \frac{2}{m+2}$
$A\boxed{B}B$	$\tau \geq \frac{1}{m+2}$
$B\boxed{B}B$	$\tau \geq 0$

Table 1: Components of the sequential root-leaf structure in the coordination game.

2.1.2 Anti-coordination game

Consider the elementary automaton defined as follows. For interior root nodes,

$$\widetilde{s}_i(t+1) = \begin{cases} 1 & m + \widetilde{s}_{i-1}(t) + \widetilde{s}_{i+1}(t) < (m+2)\tau, \\ 0 & \widetilde{s}_{i-1}(t) + \widetilde{s}_{i+1}(t) > (m+2)\tau, \\ \widetilde{s}_i(t) & \text{otherwise.} \end{cases} \tag{14}$$

For the terminal root nodes,

$$\widetilde{s}_i(t+1) = \begin{cases} 1 & m + 1 + \widetilde{s}_{i,\text{neighbor}}(t) < (m+2)\tau, \\ 0 & \widetilde{s}_{i,\text{neighbor}}(t) > (m+2)\tau, \\ \widetilde{s}_i(t) & \text{otherwise.} \end{cases} \tag{15}$$

The fixed points of this automaton are in one-to-one correspondence with the equilibrium points of the sequential root-leaf structure.

2.1.3 Parameters of interest

Based on Tables 1 and 2, there are several relevant subdivisions of $[0,1]$ for τ. When $m = 0$, we are interested in the subdivisions $\{0\}$, $\left(0, \frac{1}{2}\right)$, $\left\{\frac{1}{2}\right\}$, $\left(\frac{1}{2}, 1\right)$, $\{1\}$. When $m = 1$, we are interested in the subdivisions $\left[0, \frac{1}{3}\right)$, $\left\{\frac{1}{3}\right\}$, $\left(\frac{1}{3}, \frac{2}{3}\right)$, $\left\{\frac{2}{3}\right\}$, and $\left(\frac{2}{3}, 1\right]$. Finally, when $m \geqslant 2$, we are interested in the subdivisions $\left[0, \frac{1}{m+2}\right)$, $\left[\frac{1}{m+2}, \frac{2}{m+2}\right)$, $\left[\frac{2}{m+2}, \frac{m}{m+2}\right]$, $\left(\frac{m}{m+2}, \frac{m+1}{m+2}\right]$, and $\left(\frac{m+1}{m+2}, 1\right]$. The specific eigenvalue calculations for each interval and each kind of game may be found in the attached notebook.

Component	Condition
$\boxed{A}A$	$\tau \geq \frac{1}{m+2}$
$\boxed{A}B$	$\tau \geq 0$
$\boxed{B}A$	$\tau \leq 1$
$\boxed{B}B$	$\tau \leq \frac{m+1}{m+2}$
$A\boxed{A}A$	$\tau \geq \frac{2}{m+2}$
$A\boxed{A}B$	$\tau \geq \frac{1}{m+2}$
$B\boxed{A}B$	$\tau \geq 0$
$A\boxed{B}A$	$\tau \leq 1$
$A\boxed{B}B$	$\tau \leq \frac{m+1}{m+2}$
$B\boxed{B}B$	$\tau \leq \frac{m}{m+2}$

Table 2: Components of the sequential root-leaf structure in an anti-coordination game.

3 Star

3.1 Analysis of the number of equilibrium states

Define (A_r, A_l) as the state of the star graph, where $A_r \in \{0,1\}$ and $A_l \in \{0,1,\cdots,N-1\}$ correspond to the number of individual choosing A at the root node and leaf nodes respectively. There are only two equilibrium states for the star graph, that is, $(0,0)$, $(1,N-1)$ in the coordination game and $(0,N-1)$ and $(1,0)$ in the anti-coordination game, which means all individuals at leaf nodes must adopt the same(opposite) strategy in the coordinating(anti-coordinating) game.

Take the coordination game as an example. Suppose there exists another equilibrium state except $(0,0)$ and $(1,N-1)$. Thus, individuals adopting strategy A and B coexist at leaf nodes. If the individual at the root node uses strategy A, then for the individual i who adopts strategy B at leaf nodes, we obtain

$$n_i(t) = 1 > \tau_i k_i = \tau_i, \tag{16}$$

which means individual i tend to switch to strategy A. If the individual at root node uses strategy B, then for the individual i who adopts strategy A at leaf nodes, we have

$$n_i(t) = 0 < \tau_i k_i = \tau_i, \tag{17}$$

which means individual i tend to switch to strategy B. Therefore the current state of the system is not an equilibrium state and there are only two equilibrium states $(0,0)$ and $(1,N-1)$ in the coordination game.

3.2 Analysis of the equilibrium time

Let $p_{i\to j}^{h\to l}$ represent the probability of moving from state (h,i) to state (l,j). The average time until absorption, starting from state (h,i), is denoted by T_i^h. Then for the coordination game, we obtain

$$p_{i\to i-1}^{0\to 0} = \frac{i}{N}, \tag{18}$$

$$p_{i\to i+1}^{1\to 1} = \frac{N-i-1}{N}, \tag{19}$$

$$p_{i\to i}^{0\to 1} = \frac{1-H(\tau(N-1)-i)}{N}, \tag{20}$$

$$p_{i\to i}^{1\to 0} = \frac{1-H(i-\tau(N-1))}{N}, \tag{21}$$

where $H(\cdot)$ is the Heaviside step function and N is the number of nodes on the star. Meanwhile, we have

$$T_i^0 = p_{i\to i-1}^{0\to 0}T_{i-1}^0 + p_{i\to i}^{0\to 1}T_i^1 + (1-p_{i\to i-1}^{0\to 0} - p_{i\to i}^{0\to 1})T_i^0 + 1, \tag{22}$$

$$T_i^1 = p_{i\to i+1}^{1\to 1}T_{i+1}^1 + p_{i\to i}^{1\to 0}T_i^0 + (1-p_{i\to i+1}^{1\to 1} - p_{i\to i}^{1\to 0})T_i^1 + 1. \tag{23}$$

The boundary conditions are set as $T_0^0 = 0$ and $T_{N-1}^1 = 0$. Rearranging the equations, we obtain

$$T_i^0 = \frac{p_{i\to i-1}^{0\to 0}}{p_{i\to i-1}^{0\to 0}+p_{i\to i}^{0\to 1}}T_{i-1}^0 + \frac{p_{i\to i}^{0\to 1}}{p_{i\to i-1}^{0\to 0}+p_{i\to i}^{0\to 1}}T_i^1 + \frac{1}{p_{i\to i-1}^{0\to 0}+p_{i\to i}^{0\to 1}}, \tag{24}$$

$$T_i^1 = \frac{p_{i-1\to i}^{1\to 1}+p_{i-1\to i-1}^{1\to 0}}{p_{i-1\to i}^{1\to 1}}T_{i-1}^1 - \frac{p_{i-1\to i-1}^{1\to 0}}{p_{i-1\to i}^{1\to 1}}T_{i-1}^0 - \frac{1}{p_{i-1\to i}^{1\to 1}}. \tag{25}$$

Let $\pi_{i\to j}^{h\to l}$ represent the probability of transition, given that the system does not stay in its current state, namely

$$\pi_{i\to i+1}^{1\to 1} = 1-\pi_{i\to i}^{1\to 0} = \frac{p_{i\to i+1}^{1\to 1}}{p_{i\to i+1}^{1\to 1}+p_{i\to i}^{1\to 0}}, \tag{26}$$

$$\pi_{i\to i}^{0\to 1} = 1-\pi_{i\to i-1}^{0\to 0} = \frac{p_{i\to i}^{0\to 1}}{p_{i\to i}^{0\to 1}+p_{i\to i-1}^{0\to 0}}. \tag{27}$$

Thus we obtain

$$T_i^0 = \pi_{i\to i-1}^{0\to 0} T_{i-1}^0 + \pi_{i\to i}^{0\to 1} T_i^1 + \frac{1}{p_{i\to i-1}^{0\to 0} + p_{i\to i}^{0\to 1}}, \tag{28}$$

$$T_i^1 = \frac{1}{\pi_{i-1\to i}^{1\to 1}} T_{i-1}^1 - \frac{\pi_{i-1\to i-1}^{1\to 0}}{\pi_{i-1\to i}^{1\to 1}} T_{i-1}^0 - \frac{1}{\pi_{i-1\to i}^{1\to 1}(p_{i-1\to i}^{1\to 1} + p_{i-1\to i-1}^{1\to 0})}. \tag{29}$$

Solving inductively, we get

$$T_i^1 = A(1,i)T_1^1 - \sum_{l=2}^{i} A(l,i)B(l), \tag{30}$$

where

$$A(l,m) = 1 + \sum_{j=1}^{m-1} \pi_{j\to j}^{1\to 0} \prod_{k=l}^{j} \frac{\pi_{k\to k-1}^{0\to 0}}{\pi_{k\to k+1}^{1\to 1}}, \tag{31}$$

$$B(l) = \frac{\pi_{l-1\to l-1}^{1\to 0}}{\pi_{l-1\to l}^{1\to 1}} \sum_{j=1}^{l-1} \left(\frac{\prod_{k=j+1}^{l-1} \pi_{k\to k-1}^{0\to 0}}{p_{j\to j-1}^{0\to 0} + p_{j\to j}^{0\to 1}}\right) + \frac{1}{p_{l-1\to l}^{1\to 1}}. \tag{32}$$

And for the anti-coordination game, we have

$$p_{i\to i+1}^{0\to 0} = \frac{N-i-1}{N}, \tag{33}$$

$$p_{i\to i-1}^{1\to 1} = \frac{i}{N}, \tag{34}$$

$$p_{i\to i}^{1\to 0} = \frac{1 - H(\tau(N-1) - i)}{N}, \tag{35}$$

$$p_{i\to i}^{0\to 1} = \frac{1 - H(i - \tau(N-1))}{N}, \tag{36}$$

where $H(\cdot)$ is the Heaviside step function and N is the number of nodes on the star. Similarly, we have

$$T_i^0 = p_{i\to i+1}^{0\to 0} T_{i+1}^0 + p_{i\to i}^{0\to 1} T_i^1 + (1 - p_{i\to i+1}^{0\to 0} - p_{i\to i}^{0\to 1}) T_i^0 + 1, \tag{37}$$

$$T_i^1 = p_{i\to i-1}^{1\to 1} T_{i-1}^1 + p_{i\to i}^{1\to 0} T_i^0 + (1 - p_{i\to i-1}^{1\to 1} - p_{i\to i}^{1\to 0}) T_i^1 + 1. \tag{38}$$

with boundary conditions $T_0^1 = 0$ and $T_{N-1}^0 = 0$. And the conditional transition probability are

$$\pi_{i\to i+1}^{0\to 0} = 1 - \pi_{i\to i}^{0\to 1} = \frac{p_{i\to i+1}^{0\to 0}}{p_{i\to i+1}^{0\to 0} + p_{i\to i}^{0\to 1}}, \tag{39}$$

$$\pi_{i\to i}^{1\to 0} = 1 - \pi_{i\to i-1}^{1\to 1} = \frac{p_{i\to i}^{1\to 0}}{p_{i\to i}^{1\to 0} + p_{i\to i-1}^{1\to 1}}. \tag{40}$$

Rearranging the equations, we obtain

$$T_i^1 = \pi_{i\to i-1}^{1\to 1} T_{i-1}^1 + \pi_{i\to i}^{1\to 0} T_i^0 + \frac{1}{p_{i\to i}^{1\to 0} + p_{i\to i-1}^{1\to 1}}, \tag{41}$$

$$T_i^0 = \frac{1}{\pi_{i-1\to i}^{0\to 0}} T_{i-1}^0 - \frac{\pi_{i-1\to i-1}^{0\to 1}}{\pi_{i-1\to i}^{0\to 0}} T_{i-1}^1 - \frac{1}{\pi_{i-1\to i}^{0\to 0}(p_{i-1\to i}^{0\to 0} + p_{i-1\to i-1}^{0\to 1})} \tag{42}$$

Similarly, by solving inductively, we have

$$T_i^0 = C(1,i)T_1^0 - \sum_{l=2}^{i} C(l,i)D(l), \tag{43}$$

where

$$C(l,m) = 1 + \sum_{j=1}^{m-1} \pi_{j\to j}^{0\to 1} \prod_{k=l}^{j} \frac{\pi_{k\to k-1}^{1\to 1}}{\pi_{k\to k+1}^{0\to 0}}, \tag{44}$$

$$D(l) = \frac{\pi_{l-1\to l-1}^{0\to 1}}{\pi_{l-1\to l}^{0\to 0}} \sum_{j=1}^{l-1} \left(\frac{\prod_{k=j+1}^{l-1} \pi_{k\to k-1}^{1\to 1}}{p_{j\to j-1}^{1\to 1} + p_{j\to j}^{1\to 0}}\right) + \frac{1}{p_{l-1\to l}^{0\to 0}}. \tag{45}$$

Note that the equilibrium time calculated in this section may not equal the strategy switch number because an individual may not change his strategy after being activated.

4 Bipartite graph

Here we consider the complete bipartite graph where every node at the first(right) subset is connected to all the nodes at the second(left) subset. In our complete bipartite graph model, each node (individual) at the left subset has the same threshold, denoted by τ_l, and the count of these nodes is n_l. Conversely, the threshold of nodes at the right subset is τ_r, with the number of nodes denoted as n_r. Define the pair (A_l, A_r) to represent the state of a bipartite graph, where A_l, taking values from $0, 1, \ldots, n_l$, denotes the number of A-individuals at the left subset, and A_r, within the range $0, 1, \ldots, n_r$, indicates the number of A-individuals at the right subset. For individual i at the left subset, n_i equals A_r and $k_i = n_r$. Similarly, for individual j at the right subset, $n_j = A_l$ and $k_j = n_l$. The total number of nodes in the graph, which equates to the total number of individuals, is given by $N = n_l + n_r$.

4.1 Analysis of the number of equilibrium states

In the coordination game, we find two(or three) equilibrium states:$(0,0)$, (n_l,n_r) and $(\tau_r n_l, \tau_l n_r)$ for the system, where the third equilibrium state $(\tau_r n_l, \tau_l n_r)$ is valid only if $\tau_r n_l$ and $\tau_l n_r$ are integers. The equilibrium states $(n_l,0)$ and $(0,n_r)$ are readily verifiable. Taking $(n_l,0)$ as an example, the left individuals, satisfying $n_i = 0 < \tau_l k_i = \tau_l n_r$, choose strategy A. In contrast, the right individuals, with $n_j = n_l > \tau_r k_j = \tau_r n_l$, adopt strategy B. Thus, all individuals on the left select strategy A, and those on the right select strategy B, ensuring the equilibrium of the system. The case of $(0,n_r)$ follows similarly. In addition, if $\tau_l n_r$ and $\tau_r n_l$ are both integers, then $(\tau_r n_l, \tau_l n_r)$ is also an equilibrium state of the system. Because according to the best-response dynamics, for both the left and right individuals, $n_i = \tau_l k_i = \tau_l n_r$ and $n_j = \tau_r k_j = \tau_r n_l$ holds, all individuals keep the current strategy unchanged. It is important to note that the equilibrium state $(\tau_r n_l, \tau_l n_r)$ is unstable. Specifically, it cannot be reached from any adjacent state. This implies that any small perturbation can drive the system completely away from this equilibrium.

Here we explain why the equilibrium state $(\tau_r n_l, \tau_l n_r)$ is unstable. It is easy to verify that starting from the four equilibrium states adjacent to it, the system never reaches the equilibrium state $(\tau_r n_l, \tau_l n_r)$, i.e

$$
\begin{aligned}
(\tau_r n_l - 1, \tau_l n_r) &\rightarrow (\tau_r n_l - 1, \tau_l n_r + 1), \\
(\tau_r n_l + 1, \tau_l n_r) &\rightarrow (\tau_r n_l + 1, \tau_l n_r - 1), \\
(\tau_r n_l, \tau_l n_r - 1) &\rightarrow (\tau_r n_l + 1, \tau_l n_r - 1), \\
(\tau_r n_l, \tau_l n_r + 1) &\rightarrow (\tau_r n_l - 1, \tau_l n_r + 1).
\end{aligned}
$$

Concisely, in the state $(\tau_r n_l - 1, \tau_l n_r)$, left individuals, for whom $n_i = A_r = \tau_l n_r = \tau_l k_i$, tend to remain their current strategy. In contrast, right individuals with $n_i = A_l = \tau_r n_l - 1 < \tau_r n_l$ are likely to adopt strategy A when activated. This invariably shifts the system from $(\tau_r n_l - 1, \tau_l n_r)$ to $(\tau_r n_l - 1, \tau_l n_r + 1)$. Hence, under the assumption that a maximum of one individual updates its strategy per time step, the equilibrium state $(\tau_r n_l, \tau_l n_r)$ can never be reached unless it is the initial state of the system. The mechanism is similar in the remaining cases.

Similarly, in the anti-coordination game, the system achieves equilibrium at the states $(n_l,0)$ and $(0,n_r)$, representing scenarios where all individuals on one side adopt one strategy while individuals on the opposite side adopt the other. Additionally, a third potential equilibrium state, $(\tau_r n_l, \tau_l n_r)$, exists only when both $\tau_r n_l$ and $\tau_l n_r$ result in integer values.

4.2 Analysis of the equilibrium probability

Figure S18 illustrates the relationship between the number of A-individuals on the left and right sides and the equilibrium state in a bipartite graph in the anti-coordination game. The horizontal axis represents the number of A-individuals on the left and the vertical axis represents the number of A-individuals on the right, where the green(or blue) dots indicate that starting from this state, the system eventually reaches the state $(0, n_r)$ (or $(n_l, 0)$).

Let $\alpha_A = \lceil \tau_r n_l \rceil$ and $\beta_A = \lceil \tau_l n_r \rceil$. We use the notation $x(A_l, A_r)$ to represent the probability that starting from state (A_l, A_r), the system ultimately reaches the state $(n_l, 0)$. Thus we obtain

$$\begin{aligned} x(A_l,A_r) = {} & x(A_l+1,A_r)\cdot P^{l+}(A_l,A_r) + x(A_l-1,A_r)\cdot P^{l-}(A_l,A_r) \\ & + x(A_l,A_r+1)\cdot P^{r+}(A_l,A_r) + x(A_l,A_r-1)\cdot P^{r-}(A_l,A_r) \\ & + x(A_l,A_r)\cdot\{1 - P^{l+}(A_l,A_r) - P^{l-}(A_l,A_r) - P^{r+}(A_l,A_r) - P^{r-}(A_l,A_r)\}, \end{aligned} \tag{46}$$

where $P^{l+}(A_l,A_r)(P^{l-}(A_l,A_r))$ represents the probability that the number of left individuals choosing strategy A increases(decreases) by one in the next time step from the state (A_l, A_r). Similarly, $P^{l+}(A_l,A_r)$ and $P^{l-}(A_l,A_r)$ represent the probabilities for the right individuals under the same conditions.

Consequently, the probability of reaching the state $(n_l, 0)$ from any initial condition can be computed using the recurrence method

$$x(\alpha_A - m, \beta_A - k) = \sum_{t=1}^{k} \frac{\frac{(t+m-2)!}{(t-1)!}\cdot\frac{(n_l-\alpha_A+m)!}{(n_l-\alpha_A)!}\cdot\frac{(n_r-\beta_A+k)!}{(n_r-\beta_A+1+k-t)!}}{(m-1)!\cdot\frac{(N-\alpha_A-\beta_A+k+m)!}{(N-\alpha_A-\beta_A+k+1-t)!}}. \tag{47}$$

Similarly, define $y(B_l, B_r)$ as the probability that the system starts from the state where $B_l$individuals on the left choose strategy B and B_r individuals on the right choose strategy B, and finally reaches a state where all the individuals on the left choose strategy B and all the individuals on the right choose strategy A. Let $a = \lfloor \tau_r n_l \rfloor$, $b = \lfloor \tau_l n_r \rfloor$. According to the symmetry, we obtain

$$y(\alpha_B - m, \beta_B - k) = \sum_{t=1}^{k} \frac{\frac{(t+m-2)!}{(t-1)!}\cdot\frac{(n_l-\alpha_B+m)!}{(n_l-a)!}\cdot\frac{(n_r-\beta_B+k)!}{(n_r-\beta_B+1+k-t)!}}{(m-1)!\cdot\frac{(N-\alpha_B-\beta_B+k+m)!}{(N-\alpha_B-\beta_B+k+1-t)!}}. \tag{48}$$

Thus,

$$x(n_l-a+m,n_r-b+k) = 1-y(\alpha_B-m,\beta_B-k)$$
$$= 1-\sum_{t=1}^{k}\frac{\frac{(t+m-2)!}{(t-1)!}\cdot\frac{(n_l-\alpha_B+m)!}{(n_l-a)!}\cdot\frac{(n_r-\beta_B+k)!}{(n_r-\beta_B+1+k-t)!}}{(m-1)!\cdot\frac{(N-\alpha_B-\beta_B+k+m)!}{(N-\alpha_B-\beta_B+k+1-t)!}}. \tag{49}$$

Consequently, the probability of arriving at different equilibrium states from any initial condition in the anti-coordination game can be computed. This method is analogous to the one applied in the coordination game.

4.3 Analysis of the robustness

In this section, we analyze the robustness of the complete bipartite graph by adding new edges in equilibrium states. According to the properties of the complete bipartite graph, the new edges can be only added between two nodes at the same subset.

4.3.1 Coordination game

In equilibrium states $(0,0)$ and (n_l,n_r), all individuals adopts the same strategy. Consequently, the addition of new edges does not result in any changes to their strategies. Intuitively, the addition of new edges in states $(0,0)$ and (n_l,n_r) has no effect on the proportion of individuals' neighbors employing the same strategy. Actually, the proportion for any individual in states $(0,0)$ and (n_l,n_r) is 1. Take state (n_l,n_r) for an example, where all individuals adopt strategy A. After adding new edges, for any individual i, $n_i > \tau_i k_i$ holds due to $n_i = k_i$ and $\tau_i < 1$. Therefore, individual i does not change its strategy Because all of its neighbors adopt strategy A.

However, the addition of new edges exerts a considerable effect on the equilibrium state $(\tau_r n_l, \tau_l n_r)$. Here we assume $\tau_r n_l$ and $\tau_l n_r$ are both integers to ensure the existence of equilibrium state $(\tau_r n_l, \tau_l n_r)$. Randomly select two nodes to form a new edge. There are six cases (left side $A-A$, left side $B-B$, left side $A-B$, right side $A-A$, right side $B-B$, right side $A-B$). Here, the first three cases are discussed; the other three are similar.

I. **Left side $A-A$**

In this case, for the two A nodes with new edges,

$$\left.\begin{aligned} n_i &= \tau_l n_r + 1 \\ k_i &= n_r + 1 \end{aligned}\right\} \Rightarrow n_i > \tau k_i \tag{50}$$

Thus, both A nodes tend to choose the A strategy, keeping their strategy unchanged; the neighbor count and state of other nodes remain unchanged, so the system's state does not change.

II. **Left side** $B-B$

Similarly, for the B nodes with new edge,

$$\left.\begin{array}{l} n_i = \tau_l n_r \\ k_i = n_r + 1 \end{array}\right\} \Rightarrow n_i < \tau k_i \tag{51}$$

Thus, both B nodes tend to choose the B strategy, keeping their strategy unchanged; the neighbor count and state of other nodes remain unchanged, so the system's state does not change.

III. **Left side** $A-B$

In this case, one A node and one B node are chosen to be connected, labeling the A node as node one and the B node as node two. Then,

1. For node one

$$\left.\begin{array}{l} n_i = \tau_l n_r \\ k_i = n_r + 1 \end{array}\right\} \Rightarrow n_i < \tau k_i \tag{52}$$

   If node one is activated in the next time step, it switches its strategy to B.

2. For node two

$$\left.\begin{array}{l} n_i = \tau_l n_r + 1 \\ k_i = n_r + 1 \end{array}\right\} \Rightarrow n_i > \tau k_i \tag{53}$$

   If node two is activated in the next time step, it switches its strategy to A.

Moreover, for individual one and individual two in this scenario, $k_i = n_r + 1$, the situation $n_i = \tau k_i$ never occurs.

In this case, if the A node (node one) is activated first, the system definitely reaches the state $(0,0)$

Proof *Assuming node one is activated first, according to the best response dynamics, individual one switches its strategy to B. The state of the system becomes* $(\tau_r n_l - 1, \tau_l n_r)$.

At this point, for all other nodes on the left side, except for individual one and individual two, the equation $n_i = \tau_l n_r$ *holds. For individual one and individual two, the inequality* $n_i = \tau_l n_r < \tau_l k_i = \tau_l(n_r + 1)$ *holds, and these two nodes currently adopt strategy B, so all nodes on the left side keep their current state unchanged.*

For the nodes on the right side, the inequality $n_j = \tau_r n_l - 1 < \tau_r n_l$ *holds. Thus, according to best response dynamics, all nodes on the right side tend to switch their strategy to B. When any right-side node is activated, if its strategy is B, it remains unchanged; if its strategy is A, it switches to B.*

Therefore, starting from the state $(\tau_r n_l - 1, \tau_l n_r)$*, the only reachable state is* $(\tau_r n_l - 1, \tau_l n_r - 1)$

Now, for individual one and individual two, the inequality $n_i = \tau_l n_r - 1 < \tau_l k_i = \tau_l(n_r + 1)$ *holds: for the other nodes on the left side, except for individual one and individual two, the inequality* $n_i = \tau_l n_r - 1 < \tau_l k_i = \tau_l n_r$ *holds; for the nodes on the right side, the inequality* $n_i = \tau_r n_l - 1 < \tau_r n_l$ *holds. Thus, at this time and all subsequent times, for any node, we have*

$$n_i < \tau k_i. \tag{54}$$

Thus, all nodes tend to adopt strategy B. Therefore, only when the system's state reaches $(0,0)$ *does it stabilize.*

Similarly, if the B node (individual two) is first activated, the system definitely reaches the state (n_l, n_r).

Additionally, it is straightforward to calculate the probability that the system reaches the state $(0,0)$ after adding a random edge:

$$p = \frac{C^1_{\tau_r n_l} C^1_{n_l - \tau_r n_l} + C^1_{\tau_l n_r} C^1_{n_r - \tau_l n_r}}{C^2_{n_r} + C^2_{n_l}} \times \frac{1}{2}, \tag{55}$$

and the probability that the system reaches the state (n_l, n_r) after adding a random edge:

$$p = \frac{C^1_{\tau_r n_l} C^1_{n_l - \tau_r n_l} + C^1_{\tau_l n_r} C^1_{n_r - \tau_l n_r}}{C^2_{n_r} + C^2_{n_l}} \times \frac{1}{2}. \tag{56}$$

4.3.2 Anti-coordination game

First, we analyze the threshold of adding new edges for the strategy switching in equilibrium states. Consider that the initial state is $(0, n_r)$, adding an edge on the left influences only the connected nodes' strategies. Suppose a single edge addition triggers a strategy switch, then we obtain

$$n_i = n_r < \tau_l k_i = \tau_l(n_r + 1) \quad \Rightarrow \quad \tau_l > \frac{n_r}{n_r + 1}. \tag{57}$$

If a minimum of two edges must be added to the same node for a strategy switch, the following conditions must hold

$$\left.\begin{aligned} n_i = n_r \geq \tau_l(n_r + 1) \\ n_i = n_r < \tau_l k_i = \tau_l(n_r + 2) \end{aligned}\right\} \quad \Rightarrow \quad \frac{n_r}{n_r + 1} \geq \tau_l > \frac{n_r}{n_r + 2}. \tag{58}$$

In general, if K edges are required on the left for a switch

$$\frac{n_r}{n_r+K-1} \geq \tau_l > \frac{n_r}{n_r+K}. \tag{59}$$

And if K edges are required on the right

$$\frac{K-1}{n_l+K-1} \leq \tau_l < \frac{K}{n_l+K}. \tag{60}$$

Next, we discuss the evolution of the system from state $(0, n_r)$ after the strategy switch occurs. We initially consider the cases that adding a single edge on the left side can trigger a strategy switch(that is, $\tau_l > n_r/(n_r+1)$). After activation of one vertex of the new edge, the individual at this node switches its strategy to A. For another vertex of the new edge, it follows that

$$n_i = n_r + 1 > \tau_l k_i = \tau_l (n_r + 1). \tag{61}$$

For other nodes on the left, we obtain

$$n_i = n_r > \tau_l k_i = \tau_l n_r. \tag{62}$$

Clearly, when they are activated, they all remain their current strategy.

We now classify based on the value of τ_r, to discuss the evolution of the system.

I. $\tau_r \geq \frac{1}{n_l}$

In this case, for the right-side nodes,

$$n_j = 1 \leq \tau_r k_j = \tau_r n_l. \tag{63}$$

According to the best response dynamics, the right-side nodes tend to maintain their current state(when $n_j = \tau_r n_l$) or choose the A strategy(when $n_j < \tau_r n_l$). Given that the initial state of the right-side nodes is all A, combined with the analysis above, the overall system state remains unchanged. That is, after one individual at the vertex of the new edge updates its strategy to A, the system reaches a new equilibrium.

II. $\tau_r < \frac{1}{n_l}$

In this case, for the right-side nodes, it follows that

$$n_j = 1 > \tau_r k_j = \tau_r n_l. \tag{64}$$

After activation, a node on the right switches its strategy from A to B.

Obviously, when the population on both sides is sufficiently large, both conditions $\tau_l > \frac{n_r}{n_r+1}$ and $n_j = 1 > \tau_r k_j = \tau_r n_l$ are rather stringent. Thus, for most systems, the equilibrium state is not easily disrupted, or even if disrupted, does not result in large-scale changes.

Next, we discuss the final state to which the system evolves. Starting from the state $(1, n_r - 1)$, the system eventually reach equilibrium state $(n_l, 0)$.

First, we prove that $(n_l, 0)$ is the equilibrium state of the system after adding a new edge.

Proof *For nodes on the right side, the inequality $n_i = n_l > \tau_r n_l$ clearly holds, so individuals on the right-side choose the B strategy, maintaining their state unchanged.*

For nodes on the left side with no addition of the new edge, $n_i = 0 < \tau_l n_r$ clearly holds, so they adopt the A strategy, also remaining unchanged.

For nodes on the left side with the addition of the new edge, $n_i = 1 < \tau_l(n_r + 1)$ also holds, so they choose the A strategy, remaining unchanged as well.

Thus, when the system is in state $(n_l, 0)$, no matter which node is activated, they do not change their strategy. Therefore, state $(n_l, 0)$ is one equilibrium state of the system.

Next, we prove that starting from $(1, n_r - 1)$, the system eventually reach the equilibrium state $(n_l, 0)$.

Proof *We explain this from two perspectives:*

- *First, when $A_r \neq n_r$, we conclude that the individuals at left-side nodes all tend to choose strategy A.*

  For left-side nodes with the addition of the new edge, we have

$$\left.\begin{aligned} & n_i = A_r + a, \quad a \in \{0, 1\} \\ & \tau_l k_i = \tau_l(n_r + 1) > n_r \\ & A_r \leq n_r - 1 \end{aligned}\right\} \Rightarrow \tau_l k_i > n_i. \tag{65}$$

  For other nodes on the left, we get

$$\left.\begin{aligned} & n_i = A_r \\ & \tau_l k_i = \tau_l n_r > \frac{n_r^2}{n_r + 1} > n_r - 1 \\ & A_r \leq n_r - 1 \end{aligned}\right\} \Rightarrow \tau_l k_i > n_i. \tag{66}$$

  Thus, all individuals at left-side nodes tend to choose the A strategy.

- *Second, when $A_l \neq 0$, we say that all individuals at right-side nodes tend to choose the strategy B.*

For any right-side individuals, we have

$$\left.\begin{aligned} n_i &= A_l \\ \tau_r k_i &= \tau_r n_l < 1 \\ A_l &\geq 1 \end{aligned}\right\} \Rightarrow \tau_r k_i < n_i \tag{67}$$

Therefore, all right-side individuals tend to choose the B strategy.

- *In summary, starting from $(1, n_r - 1)$, $A_r \neq n_r$ and $A_l \neq 0$ are satisfied. According to the above statement, when a left-side A individual is activated, A_l remains unchanged, and when a left-side B individual is activated, A_l increases; when a right-side A individual is activated, A_r decreases, and when a left-side B individual is activated, A_r remains unchanged, thus $A_r \neq n_r$ and $A_l \neq 0$ are always met. Therefore, the system reaches equilibrium only when there are no B individuals on the left side and no A individuals on the right side, which results in the system's state remaining unchanged whichever individual is activated.*

  Therefore, starting from $(1, n_r - 1)$, the system eventually reaches state $(n_l, 0)$.

Furthermore, we analyze the scenario where the initial state is $(0, n_r)$, and adding at least f edges to a node on the left side is required to change the system state. At this time, the following inequality must hold

$$\frac{n_r}{n_r + f - 1} \geq \tau_l > \frac{n_r}{n_r + f}. \tag{68}$$

Consider the case where exactly f edges are added to a particular node on the left side, while the number of new edges added to other nodes on the left is less than f. When the node with f new edges is activated, it switches its strategy to A, changing the system's state to $(1, n_r)$.

For the other nodes on the left side, we obtain

$$\left.\begin{aligned} n_i &= n_r + 1 \\ k_i &= n_r + g_i \\ 0 \leq & g_i < f \end{aligned}\right\} \Rightarrow n_i > \tau_l (n_r + g_i). \tag{69}$$

Therefore, other nodes on the left adopt strategy B, which means they maintain their strategy unchanged.

For nodes on the right side, where $n_i = 1$, whether the strategy is updated or not depends on their threshold. We focus on the case when $\tau_r < \frac{1}{n_l}$ otherwise no individual at right-side node changes its strategy. In this case, when a node on the right is activated, it switches to strategy B, hence the system's state becomes $(1, n_r - 1)$. From this state, the subsequent evolution process of the system is discussed in the following.

For the right-side nodes, we obtain

$$\tau_r n_l < 1, \tag{70}$$

implying that as long as not all nodes on the left adopt strategy B, the right-side nodes tend to choose strategy B, continuously reducing A_r.

For the node on the left with f new edges, we have

$$n_i = A_r + a, \quad 0 \leq a \leq f, \tag{71}$$

$$\tau_l (n_r + f) > n_r. \tag{72}$$

For a node on the left with g_i new edges added:

$$n_i = A_r + a, \quad 0 \leq a \leq g_i, \tag{73}$$

$$\tau_l (n_r + g_i) > n_r \frac{n_r + g_i}{n_r + f} = n_r - n_r \frac{f - g_i}{n_r + f} > n_r - f + g_i. \tag{74}$$

It can be observed that if $n_r \geq f$ is guaranteed, then adding h edges to the left side, some of which have f edges on certain nodes while others have fewer than f, the bipartite graph evolve from the state $(0, n_r)$ to $(n_l, 0)$.(**Necessary and sufficient condition**)

Proof *I. Sufficiency*

We prove the sufficiency by contradiction. Assume if $n_r \geq f$, starting from $(0, n_r)$, the system cannot eventually reach $(n_l, 0)$, then one of the following conditions must be true in the equilibrium state: $A_r \neq 0$ or $A_l \neq n_l$.

- *If $A_r \neq 0$, according to the best response dynamics, this implies $A_l = 0$ in the equilibrium state, otherwise the state is not an equilibrium state. For the node on the left with f new edges, we get*

$$n_i = A_r + 0 = A_r \leq n_r < \tau_l (n_r + f). \tag{75}$$

  The inequity means the node tends to choose strategy A, indicating that this is not an equilibrium state, which contradicts the assumption that the system has reached an equilibrium state.

- *If $A_l \neq n_l$, then there are only two possible scenarios: $A_l = 0, A_r = n_r$ or $A_l \neq 0, A_r = 0$.*

  In the first scenario, according to best response dynamics, it is clear that this is not an equilibrium, a contradiction.

In the second scenario, there exists a node with g_i new edges and the best response dynamics lead it to choose strategy B, i.e.

$$\tau_l(n_r+g_i) \le A_r + a = a, \tag{76}$$

where $a \le g_i$ and $\tau_l(n_r+k) > \frac{n_r(n_r+k)}{n_r+f} \ge \frac{n_r^2}{n_r+f} \ge \frac{n_r^2}{n_r+n_r} \ge n_r$. Therefore, even if a reaches its maximum, it must hold that

$$n_r < k \le f, \tag{77}$$

which contradicts the condition.

Overall, the assumption does not hold, thus if $n_r \ge f$, starting from $(0,n_r)$, the system definitely reaches $(n_l,0)$.

II. Necessity

Again, we prove it by contradiction. Assume that starting from the state $(0,n_r)$, the system can reach $(n_l,0)$, but $n_r < f$.

According to best response dynamics, when the system is at equilibrium in the state $(n_l,0)$, for an individual on the left with f new edges, it must hold

$$n_i = f \le \tau_l(n_r+f). \tag{78}$$

Given the range for τ_l, we know

$$n_r + 1 \ge \tau_l(n_r+f) > n_r. \tag{79}$$

Also, since f is an integer, under the assumption, we obtain

$$f \ge n_r + 1, \tag{80}$$

which makes

$$f \ge \tau_l(n_r+f). \tag{81}$$

Under these circumstances, only if $f = \tau_l(n_r+f)$, when exactly f edges are added to the left, the system remains in the state $(0,n_r)$ with no change in strategy. This contradicts that at least one individual tends to switch his strategy, thus the assumption does not hold.

Therefore, if starting from $(0,n_r)$, the system can reach $(n_l,0)$, then it must be that $n_r \ge f$.

In conclusion, the necessary and sufficient condition is proven.

The case when the initial state is $(n_l, 0)$ follows a similar analysis.

In addition, given the initial state $(\tau_l n_r, \tau_r n_l)$, the analysis is similar to that in the coordination game. That is, randomly selecting two nodes to form a new edge, there are six cases (left side $A-A$, left side $B-B$, left side $A-B$, right side $A-A$, right side $B-B$, right side $A-B$). We mainly focus on the first three cases since the analysis for the next three cases is analogous.

I. **Left side A-A** For the two endpoints of the new edge, $n_i = \tau_l n_r + 1 > \tau_l(n_r+1)$. The initially activated node switches to strategy B. And the system eventually stabilizes at the state $(0, n_r)$.

II. **Left side B-B** For the two endpoints of the new edge, $n_i = \tau_l n_r < \tau_l(n_r+1)$. The initially activated node switches to strategy A. The system stabilizes at the state $(n_l, 0)$.

III. **Left side A-B** For the A node of the new edge, $n_i = \tau_l n_r < \tau_l(n_r+1)$, leading to no strategy change. For the B node, $n_i = \tau_l n_r + 1 > \tau_l(n_r+1)$, its strategy remains unchanged. Thus, the system state does not change.

5 Rich-club

In rich club networks, some nodes exhibit exceptionally high degrees while others display relatively low degrees. Focusing on a specific type of rich-club network where n_r rich nodes are fully connected and n_p poor nodes link to all rich nodes ($n_r + n_p = N$), the state (strategy composition) of the network can be described by a 2-tuple (A_r, A_p), where A_r and A_p denote the number of A-individuals among the rich nodes and the poor nodes respectively. Let τ_p and τ_r denote the behavioral switching threshold, respectively.

5.1 Analysis of the number of equilibrium states

5.1.1 Coordination game

There are only two equilibrium states $(0,0)$ and (n_r, n_p). The analysis is similar to that in Section 5.1.2.

5.1.2 Anti-coordination game

For poor nodes: $n_i = A_r, k_i = n_r$. Therefore, we obtain

$$s_i(t+1) = \begin{cases} A, & A_r < \tau_p n_r \\ s_i(t), & A_r = \tau_p n_r \\ B, & A_r > \tau_p n_r \end{cases} \tag{82}$$

Consider the rich nodes divided into two groups, A-rich nodes and B-rich nodes, with counts A_r and $n_r - A_r$ respectively. For A-rich nodes, we know $n_j = A_p + A_r - 1, k_j = n_r + n_p - 1$, thus

$$s_i(t+1) = \begin{cases} A, & A_p + A_r - 1 < \tau_r(n_r + n_p - 1) \\ s_i(t), & A_p + A_r - 1 = \tau_r(n_r + n_p - 1) \\ B, & A_p + A_r - 1 > \tau_r(n_r + n_p - 1) \end{cases} \tag{83}$$

For B-rich nodes, we have $n_i = A_p + A_r, k_i = n_r + n_p - 1$, therefore

$$s_i(t+1) = \begin{cases} A, & A_p + A_r < \tau_r(n_r + n_p - 1) \\ s_i(t), & A_p + A_r = \tau_r(n_r + n_p - 1) \\ B, & A_p + A_r > \tau_r(n_r + n_p - 1) \end{cases} \tag{84}$$

Based on the above equations, fifteen different scenarios can be discussed, and we find there are 11 types of equilibrium states (the number may be fewer because some equilibrium states could be merged).

I. $A_r < \tau_p n_r, A_p + A_r - 1 < \tau_r(n_p + n_r - 1)$, and $A_p + A_r > \tau_r(n_p + n_r - 1)$

poor nodes choose strategy A, A-rich nodes choose strategy A, and B-rich nodes choose strategy B.

To ensure network equilibrium, the number of A-poor nodes must be n_p, i.e., $A_p = n_p$.

For rich nodes, it must satisfy:

$$\tau_r(n_p + n_r - 1) - n_p < A_r < \tau_r(n_p + n_r - 1) - n_p + 1 \tag{85}$$

To ensure $0 \leq A_r \leq n_r$ and A_r is an integer, $\tau_r(n_p + n_r - 1)$ must not be an integer and $\tau_r > \frac{n_p - 1}{n_p + n_r - 1}$.

Thus, $A_r = \lceil \tau_r(n_p + n_r - 1) - n_p \rceil = \lfloor \tau_r(n_p + n_r - 1) - n_p + 1 \rfloor$.

To ensure $A_r < \tau_p n_r$, it must satisfy $\tau_p > \frac{\lceil \tau_r(n_p + n_r - 1) - n_p \rceil}{n_r}$.

Hence, when these conditions are met, the system can balance with n_p A-poor nodes and $\lceil \tau_r(n_p + n_r - 1) - n_p \rceil$ A-rich nodes, establishing an equilibrium state of $(\lceil \tau_r(n_p + n_r - 1) - n_p \rceil, n_p)$.

II. $A_r < \tau_p n_r, A_p + A_r - 1 = \tau_r(n_p + n_r - 1)$, and $A_p + A_r > \tau_r(n_p + n_r - 1)$

poor nodes choose strategy A, A-rich nodes maintain their strategy, and B-rich nodes choose strategy B.

To make these conditions valid while ensuring $0 \leq A_r \leq n_r$ and A_r is an integer, it must be ensured that:

- $\tau_r(n_p+n_r-1)$ is an integer.
- $\tau_r \geq \frac{n_p-1}{n_p+n_r-1}$.
- $\tau_p > \frac{\tau_r(n_p+n_r-1)-n_p+1}{n_r}$.

Under these conditions, the system can balance with n_p A-poor nodes and $\tau_r(n_p+n_r-1)-n_p+1$ A-rich nodes, establishing an equilibrium state of $(\tau_r(n_p+n_r-1)-n_p+1, n_p)$.

III. $A_r < \tau_p n_r, A_p+A_r-1 < \tau_r(n_p+n_r-1)$, and $A_p+A_r = \tau_r(n_p+n_r-1)$

poor nodes choose strategy A, A-rich nodes choose strategy A, and B-rich nodes maintain their strategy.

To make these conditions valid while ensuring $0 \leq A_r \leq n_r$ and A_r is an integer, it must be ensured that

- $\tau_r(n_p+n_r-1)$ is an integer.
- $\tau_r \geq \frac{n_p}{n_p+n_r-1}$.
- $\tau_p > \frac{\tau_r(n_p+n_r-1)-n_p}{n_r}$.

Under these conditions, the system can balance with n_p A-poor nodes and $\tau_r(n_p+n_r-1)-n_p$ A-rich nodes, establishing an equilibrium state of $(\tau_r(n_p+n_r-1)-n_p, n_p)$.

IV. $A_r < \tau_p n_r, A_p+A_r-1 > \tau_r(n_p+n_r-1), A_p+A_r > \tau_r(n_p+n_r-1)$

poor nodes choose strategy A, A-rich nodes choose strategy B, and B-rich nodes also choose strategy B.

To achieve system equilibrium under these conditions, all poor nodes must adopt strategy A, and all rich nodes must adopt strategy B, i.e., $A_p = n_p$, $A_r = 0$. Substituting these values into the inequalities yields:

- $\tau_r < \frac{n_p-1}{n_p+n_r-1}$
- $\tau_p > 0$ (obviously)

With these conditions met, the system can reach equilibrium with n_p poor nodes choosing A and 0 rich nodes choosing A, resulting in an equilibrium state of $(0, n_p)$.

V. $A_r < \tau_p n_r, A_p+A_r-1 < \tau_r(n_p+n_r-1), A_p+A_r < \tau_r(n_p+n_r-1)$

poor nodes choose strategy A, A-rich nodes choose strategy A, and B-rich nodes also choose strategy A. Therefore, the system will definitely not be in equilibrium.

VI. $A_r > \tau_p n_r, A_p + A_r - 1 < \tau_r(n_p + n_r - 1), A_p + A_r > \tau_r(n_p + n_r - 1)$

poor nodes choose strategy B, A-rich nodes choose strategy A, and B-rich nodes choose strategy B. In order for the system to balance, it must be that $A_p = 0$ and

$$\tau_r(n_p + n_r - 1) < A_r < \tau_r(n_p + n_r - 1) + 1$$

To ensure $0 \leq A_r \leq n_r$ and that A_r is an integer, and $A_r > \tau_p n_r$:

- $\tau_r(n_p + n_r - 1)$ is not an integer
- $\tau_r < \frac{n_r}{n_p + n_r - 1}$
- $\tau_p < \frac{\lceil \tau_r(n_p + n_r - 1) \rceil}{n_r}$

Under these conditions, the system can reach equilibrium with 0 poor nodes choosing A and $\lceil \tau_r(n_p + n_r - 1) \rceil$ rich nodes choosing A, resulting in an equilibrium state of $(\lceil \tau_r(n_p + n_r - 1) \rceil, 0)$.

VII. $A_r > \tau_p n_r, A_p + A_r - 1 = \tau_r(n_p + n_r - 1), A_p + A_r > \tau_r(n_p + n_r - 1)$

poor nodes choose strategy B, A-rich nodes remain unchanged, and B-rich nodes choose strategy B.

To ensure that the above conditions hold, while also ensuring that $0 \leq A_r \leq n_r$ and that A_r is an integer, the following must be satisfied:

- $\tau_r(n_p + n_r - 1)$ is an integer
- $\tau_r \leq \frac{n_r - 1}{n_p + n_r - 1}$
- $\tau_p < \frac{\tau_r(n_p + n_r - 1) + 1}{n_r}$

With these conditions, the system can reach equilibrium with 0 poor nodes choosing A and $\tau_r(n_p + n_r - 1) + 1$ rich nodes choosing A, resulting in an equilibrium state of $(\tau_r(n_p + n_r - 1) + 1, 0)$.

VIII. $A_r > \tau_p n_r, A_p + A_r - 1 < \tau_r(n_p + n_r - 1), A_p + A_r = \tau_r(n_p + n_r - 1)$

poor nodes choose strategy B, A-rich nodes choose strategy A, and B-rich nodes remain unchanged.

To ensure these conditions hold, while also ensuring that $0 \leq A_r \leq n_r$ and that A_r is an integer, the following must be satisfied:

- $\tau_r(n_p + n_r - 1)$ is an integer
- $\tau_r \leq \frac{n_r}{n_p + n_r - 1}$
- $\tau_p < \frac{\tau_r(n_p + n_r - 1)}{n_r}$

With these conditions, the system can achieve equilibrium with 0 poor nodes choosing A and $\tau_r(n_p+n_r-1)$ rich nodes choosing A, resulting in an equilibrium state of $(\tau_r(n_p+n_r-1), 0)$.

IX. $A_r > \tau_p n_r, A_p+A_r-1 > \tau_r(n_p+n_r-1), A_p+A_r > \tau_r(n_p+n_r-1)$

poor nodes choose strategy B, A-rich nodes choose strategy B, and B-rich nodes also choose strategy B. Thus, the system will never be in equilibrium.

X. For the scenario where $A_r > \tau_p n_r$, $A_p+A_r-1 < \tau_r(n_p+n_r-1)$, $A_p+A_r < \tau_r(n_p+n_r-1)$: poor nodes choose strategy B, A-rich nodes choose strategy A, and B-rich nodes choose strategy A.

To achieve system equilibrium under these conditions, all poor nodes must adopt strategy B, and all rich nodes must adopt strategy A, i.e., $A_p = 0$, $A_r = n_r$. Substituting these values into the inequalities yields:

- $\tau_r > \frac{n_r}{n_p+n_r-1}$
- $\tau_p < 1$ (obviously)

With these conditions met, the system can reach equilibrium with 0 poor nodes choosing A and n_r rich nodes choosing A, resulting in an equilibrium state of $(n_r, 0)$.

XI. $A_r = \tau_p n_r, A_p+A_r-1 < \tau_r(n_p+n_r-1), A_p+A_r > \tau_r(n_p+n_r-1)$

poor nodes maintain their current strategy, A-rich nodes choose strategy A, and B-rich nodes choose strategy B.

At the same time, $A_r = \tau_p n_r$ and $\tau_r(n_p+n_r-1)-\tau_p n_r < A_p < \tau_r(n_p+n_r-1)-\tau_p n_r+1$

To ensure $0 \le A_p \le n_p$ and that all conditions are satisfied, it must be ensured that:

- $\tau_r(n_p+n_r-1)$ is not an integer
- $\tau_p n_r$ is an integer
- $\frac{\tau_p n_r-1}{n_p+n_r-1} < \tau_r < \frac{n_p+\tau_p n_r}{n_p+n_r-1}$ or $\frac{\tau_r(n_p+n_r-1)-n_p}{n_r} < \tau_p < \frac{\tau_r(n_r+n_p-1)+1}{n_r}$

Under these conditions, the system can achieve equilibrium with $\lceil \tau_r(n_p+n_r-1)-\tau_p n_r \rceil$ poor nodes choosing A and $\tau_p n_r$ rich nodes choosing A, resulting in an equilibrium state of $(\tau_p n_r, \lceil \tau_r(n_p+n_r-1)-\tau_p n_r \rceil)$.

XII. $A_r = \tau_p n_r, A_p+A_r-1 = \tau_r(n_p+n_r-1), A_p+A_r > \tau_r(n_p+n_r-1)$

poor nodes maintain their current strategy, A-rich nodes remain unchanged, and B-rich nodes choose strategy B.

To ensure $0 \le A_p \le n_p$ and that all conditions are satisfied, the following must be ensured:

- $\tau_r(n_p+n_r-1)$ is an integer
- $\tau_p n_r$ is an integer
- $\frac{\tau_p n_r-1}{n_p+n_r-1} \leq \tau_r \leq \frac{n_p+\tau_p n_r-1}{n_p+n_r-1}$ or $\frac{\tau_r(n_p+n_r-1)-n_p+1}{n_r} \leq \tau_p \leq \frac{\tau_r(n_r+n_p-1)+1}{n_r}$

With these conditions met, the system can achieve equilibrium with $\tau_r(n_p+n_r-1)-\tau_p n_r+1$ poor nodes choosing A and $\tau_p n_r$ rich nodes choosing A, resulting in an equilibrium state of $(\tau_p n_r, \tau_r(n_p+n_r-1)-\tau_p n_r+1)$.

XIII. $A_r = \tau_p n_r, A_p + A_r - 1 < \tau_r(n_p+n_r-1), A_p + A_r = \tau_r(n_p+n_r-1)$

poor nodes maintain their current strategy, A-rich nodes choose strategy A, and B-rich nodes remain unchanged.

To ensure $0 \leq A_p \leq n_p$ and that all conditions are satisfied, the following must be ensured:

- $\tau_r(n_p+n_r-1)$ is an integer
- $\tau_p n_r$ is an integer
- $\frac{\tau_p n_r}{n_p+n_r-1} \leq \tau_r \leq \frac{n_p+\tau_p n_r}{n_p+n_r-1}$ or $\frac{\tau_r(n_p+n_r-1)-n_p}{n_r} \leq \tau_p \leq \frac{\tau_r(n_r+n_p-1)}{n_r}$

With these conditions met, the system can achieve equilibrium with $\tau_r(n_p+n_r-1)-\tau_p n_r$ poor nodes choosing A and $\tau_p n_r$ rich nodes choosing A, resulting in an equilibrium state of $(\tau_p n_r, \tau_r(n_p+n_r-1)-\tau_p n_r)$.

XIV. $A_r = \tau_p n_r, A_p + A_r - 1 > \tau_r(n_p+n_r-1), A_p + A_r > \tau_r(n_p+n_r-1)$

The system is not in equilibrium.

XV. $A_r = \tau_p n_r, A_p + A_r - 1 < \tau_r(n_p+n_r-1), A_p + A_r < \tau_r(n_p+n_r-1)$

The system is not in equilibrium.

5.2 Analysis of the robustness

5.2.1 Coordination game

There are only two equilibrium states $(0,0)$ and (n_r, n_p). Obviously, adding an edge does not have any effect.

5.2.2 Anti-coordination game

For all the conditions that can make the system in equilibrium, where I, II, III, and IV are similar to VI, VII, VIII, and X, only I, II, III, and IV are discussed, and the analysis last four are similar to them; XI, XII, XIII is the same case, we just consider XI.

- For Scenario I, where the equilibrium state is $A_r = \lceil \tau_r(n_p+n_r-1)-n_p \rceil, A_p = n_p$, the conditions $\tau_r > \frac{n_p-1}{n_p+n_r-1}$ and $\tau_p > \frac{\lceil \tau_r(n_p+n_r-1)-n_p \rceil}{n_r}$ must hold. Considering the rich-club property, where all rich nodes are already connected to every other node, new edges can only be added between two poor nodes.

  In this situation, after adding an edge, the system's state change and the poor nodes' threshold τ_p are crucial. Examining the addition of a single edge, if an edge is added between two poor nodes, the system remains unchanged if $\tau_p \geq \frac{\lceil \tau_r(n_p+n_r-1)-n_p+1 \rceil}{n_r+1}$. If $\tau_p < \frac{\lceil \tau_r(n_p+n_r-1)-n_p+1 \rceil}{n_r+1}$, according to best response dynamics, one endpoint of the new edge switches to strategy B, causing a B- rich node to switch to strategy A, i.e., $A_r = \lceil \tau_r(n_p+n_r-1)-n_p \rceil + 1, A_p = n_p - 1$. Subsequently, poor nodes not connected by the new edge change their strategies according to best response dynamics, and for every poor node that switches to strategy B, a poor node switches to strategy A until equilibrium is reached. Eventually, the system evolves into a new equilibrium state, where:

$$A_r^* = \min\{n_r, \lceil \tau_r(n_p+n_r-1) \rceil\} \tag{86}$$

$$A_p^* = 0 \tag{87}$$

  In more complex scenarios, the following conclusion can be drawn: if $\frac{\lceil \tau_r(n_p+n_r-1)-n_p \rceil + f - 1}{n_r+f-1} < \tau_p < \frac{\lceil \tau_r(n_p+n_r-1)-n_p \rceil + f}{n_r+f}$, and if at least f poor nodes (if fewer, then only those nodes and a corresponding number of rich nodes change) have been added at least f edges each (these nodes do not connect to each other), the final equilibrium state of the system will be:

$$A_r^* = \min\{n_r, \lceil \tau_r(n_p+n_r-1) \rceil\} \tag{88}$$

$$A_p^* = 0 \tag{89}$$

- For Scenario II, where the equilibrium state is $A_r = \tau_r(n_p+n_r-1) - n_p + 1, A_p = n_p$), it follows that:

$$\tau_r(n_p+n_r-1) = n_p, \cdots, n_p+n_r-2 \tag{90}$$

$$\tau_p > \frac{\tau_r(n_p+n_r-1)-n_p+1}{n_r} = \frac{k}{n_r} \tag{91}$$

  Similar conclusions can be drawn, indicating when $\frac{k+f-1}{n_r+f-1} \leq \tau_p < \frac{k+f}{n_r+f}$, if $f+1$ poor nodes (if fewer, then only those nodes and a corresponding number minus one of rich nodes change) have been added at least f (more than 1) edges each (these nodes do not connect to each other), then the final equilibrium state of the system will be:

$$A_r^* = \min\{n_r, \lceil \tau_r(n_p+n_r-1) - 1 \rceil\} \tag{92}$$

$$A_p = 0 \tag{93}$$

- Scenario III is similar to I.
- Scenarios VI, VII, VIII, and X follow similar analysis methods.
- For Scenario IV, when the equilibrium state is $A_r^* = 0, A_p^* = n_p$, and it satisfies $0 < \tau_r < \frac{n_p-1}{n_p+n_r-1}$, the following holds:

  If m poor nodes have been added f edges (these nodes do not connect to each other), the dynamics are influenced by:

  - If $\tau_p > \frac{f}{n_r+f}$, there is no change.
  - If $\frac{f-1}{n_r+f-1} \leq \tau_p < \frac{f}{n_r+f}$ and if $\frac{n_p-k}{n_p+n_r-1} < \tau_r \leq \frac{n_p-k+1}{n_p+n_r-1}$, and provided $m-k+1 \geq f, n_r \geq f$, then the final equilibrium state of the system is:

$$A_p = 0 \tag{94}$$

$$A_r = \min\{n_r, n_p - k + 1\} \tag{95}$$

- For Scenario XI, the equilibrium state is characterized by $\tau_p n_r = k, k = 1, 2, \cdots, n_r - 1$, $\frac{k-1}{n_p+n_r-1} < \tau_r < \frac{k+n_p}{n_p+n_r-1}$. Given this equilibrium state, both A and strategy B agents exist externally, and since this equilibrium is inherently unstable, the following conclusions can be drawn:

  - If the endpoints of a new edge have different strategies, the system remains unchanged.
  - If both endpoints of a new edge are A-individuals, the final equilibrium state will be:

$$A_p = 0 \tag{96}$$

$$A_r = \min\{n_r, \tau_r \lceil n_p + n_r - 1 \rceil\} \tag{97}$$

  - If both endpoints of a new edge are B-individuals, the final equilibrium state will be:

$$A_p = n_p \tag{98}$$

$$A_r = \max\{0, \tau_r \lfloor n_p + n_r - 1 \rfloor - n_p + 1\} \tag{99}$$

6 Supplementary Discussion

6.1 The relationship between behavioral switching threshold τ and the average frequency of strategies in equilibrium states

We analyze the average frequency of strategy A, denoted by

$$\bar{s} = \sum_{\mathbf{s}\in\mathbb{S}_G^{\tau+}} p(\mathbf{s})\frac{||\mathbf{s}||}{N}, \tag{100}$$

or

$$\bar{s} = \sum_{\mathbf{s}\in\mathbb{S}_G^{\tau-}} p(\mathbf{s})\frac{||\mathbf{s}||}{N}, \tag{101}$$

where $\mathbb{S}_G^{\tau+}$ (or $\mathbb{S}_G^{\tau-}$) is the set of network G's equilibrium states in a coordinating (or anti-coordinating) game when the value of behavioral switching threshold equals τ, and $p(\mathbf{s})$ is the probability that the system goes into the absorbing state $\mathbf{s}$ when the initial state is chosen from a uniform distribution.

Figure S19**ab** shows that the frequency of strategy is quite close to 1 when τ is less than 0.5, which means the favored strategy dominates networks in coordination games. Figure S19**cd** illustrates that the strategy frequency is quite closer to 0.5 when τ is less than 0.5, meaning two strategies coexist in anti-coordination games. The above conclusion conforms to what we discussed in the main contents.

6.2 The Role of Noise in Decision-Making

Now, consider the decision-making process incorporates a stochastic component, which is fundamental to understanding the nature of the system's equilibria. This stochasticity could be formally modeled through the concept of bounded rationality. The probability that an individual i adopts strategy A is governed by a logistic choice function:

$$p_i^A = \frac{\exp(\beta u_i^A)}{\exp(\beta u_i^A)+\exp(\beta u_i^B)} = \frac{1}{1+\exp[\beta(u_i^B - u_i^A)]}, \tag{102}$$

where u_i^A and u_i^B represent the payoffs for choosing strategies A and B, respectively, and the parameter $\beta \in [0,\infty)$ quantifies the level of individual rationality. The parameter β determines the sensitivity of an individual to payoff differences. A high value of β signifies high rationality, where even small payoff advantages are detected and acted upon. Conversely, as $\beta \to 0$, choices become nearly random.

From a stochastic process perspective, now the equilibrium states are not strictly absorbing. Once the system reaches such a state, there is always a non-zero probability of transitioning away from it. This behavior contrasts with many standard imitation-based learning models. In

imitation dynamics, noise often does not alter the fundamental properties of the corresponding Markov chains, which typically remain non-recurrent. In such cases, the system will inevitably become trapped in an absorbing state unless an explicit exploration mechanism (e.g., mutation) is introduced.

This formulation allows for a direct interpretation in terms of noise intensity by defining a noise parameter $\eta = 1/\beta$. Under this interpretation, low noise ($\eta \to 0$) corresponds to high rationality, enabling individuals to reliably choose the optimal strategy, while high noise ($\eta \to \infty$) increases the likelihood of erroneous choices. A key consequence of this formulation is that for any finite level of noise, the system's equilibria are best described as metastable states. Although no state is strictly absorbing, the escape time required for the system to transition from one equilibrium's basin of attraction to another can be very large (Fig. S21). Therefore, once the system settles into an equilibrium, it will primarily fluctuate around that state for extended periods.

6.3 Comparison to other updating rules

Consider the DB process when the selection intensity is infinite. The probability that node j is occupied by the offspring of i is

$$p_{ij} = \begin{cases} \dfrac{k_{ij}}{\sum_{\ell \in M_j} k_{\ell j}}, & i \in M_j, \\ 0, \quad i \notin M_j, \end{cases} \tag{103}$$

where $M_j = \{\ell | u_\ell = \max_{n \in \mathcal{N}_j} u_n\}$. Therefore, $\mathbf{s} = (s_1, s_2, \cdots, s_N)^T$ is an equilibrium state if and only if

- $\forall i \in \mathcal{N}, \forall j, \ell \in M_i, \quad s_j = s_\ell,$
- $\forall i \in \mathcal{N}, \forall j \in M_i, \quad s_j = s_i.$

Here we consider two types of regular graphs: ring lattice graphs and cyclic grid graphs. For the coordination game

$$\begin{array}{cc} & \begin{array}{cc} A & B \end{array} \\ \begin{array}{c} A \\ B \end{array} & \begin{pmatrix} 1,1 & 0,0 \\ 0,0 & d,d \end{pmatrix}, \end{array} \tag{104}$$

in ring lattice graphs ($N = 16, 20$), there are only two equilibrium states: $\mathbf{s} = \mathbf{0}$ or $\mathbf{s} = \mathbf{1}$. In cyclic grid graphs, the number of equilibrium states is determined by the value of d, as shown below

d	Equilibrium number
0.1	2
0.3	98
0.5	18
0.8	114
1.1	114
1.3	114
1.5	114
1.6	650
1.8	650
2.0	18
2.5	18
2.8	18
3.0	18
3.2	98
3.8	98

Table 3: Non-linear relationship between payoff and equilibrium state number under DB updating. Parameters: $N = 16$, $\bar{k} = 4$.

For the anti-coordination game

$$\begin{array}{c} \\ A \\ B \end{array}\begin{array}{c} \begin{array}{cc} A & B \end{array} \\ \begin{pmatrix} 0,0 & 1,c \\ c,1 & 0,0 \end{pmatrix} \end{array}, \tag{105}$$

both in ring lattice and cyclic grid graphs ($N = 9, 16$), there are only two equilibrium states: $\mathbf{s} = \mathbf{0}$ or $\mathbf{s} = \mathbf{1}$. The results are quite different from coordinating or anti-coordinating systems.

Consider the IM process when the selection intensity is infinite. The probability that node j is occupied by the offspring of i is

$$p_{ij} = \begin{cases} \dfrac{k_{ij}}{\sum_{\ell \in M_j} k_{\ell j}}, & i \in M_j, \\ 0, \quad i \notin M_j, \end{cases} \tag{106}$$

where $M_j = \{\ell | u_\ell = \max_{n \in \mathscr{N}_j \cup \{j\}} u_n\}$. Therefore, $\mathbf{s} = (s_1, s_2, \cdots, s_N)^T$ is an equilibrium state if and only if $\forall i \in \mathscr{N}, \forall j \in M_i, s_j = s_i$ holds.

Meanwhile, consider the BD process when the selection intensity is infinite. The probability that node j is occupied by the offspring of i is

$$p_{ij} = \begin{cases} \dfrac{k_{ij}}{\sum_{\ell \in \mathscr{N}} k_{i\ell}}, & i \in M, \\ 0, \quad i \notin M, \end{cases} \tag{107}$$

where $M = \{\ell | u_\ell = \max_{n \in \mathcal{N}} u_n\}$. Therefore, $\mathbf{s} = (s_1, s_2, \cdots, s_N)^T$ is an equilibrium state if and only if $\forall i \in M, \forall j \in \mathcal{N}_i, s_j = s_i$ holds.

Different imitation rules makes the system dynamics diverse. However, all imitation rules are sensitive to the perturbation (like the payoff structure), as shown in Fig. 20, while systems under coordination/anti-coordination dynamics are robust to those perturbation. This is one of the advantages of coordination/anti-coordination dynamics. The above analysis reflects the differences between systems based on imitation and those based on coordination or anti-coordination.

Abbreviation	N	E	$\bar{k}$	Description
7G	29	240	16.55	Friendship among 7th grade students [1]
BE	28	131	9.36	Interaction among beetles [2]
BC	25	90	7.20	CEO club membership network [3]
FE	32	266	16.63	Personal message exchange [4]
KC	34	78	4.59	Zachary's karate club friendship [5]
MX	31	105	6.77	Mexican political elites network [6]
MZ	23	105	9.13	Interaction among zebra [7]
MB	26	222	17.08	Interaction among bison [8]
MC	28	205	14.64	Interaction among cattle [9]
MK	17	91	10.71	Kangaroo social network [10]
MSa	18	126	14.00	Relationships among monks [11]
MSh	28	235	16.79	Relationships between bighorn sheep [12]
PV	22	39	3.55	Social network in a Papuan village [13]
RA	16	42	5.25	Raccoon social network [2]
RM	16	69	8.63	Macaque social network [14]
SM	17	70	8.24	Spider monkey interaction network [2]
TSF	39	158	8.10	Tailor shop employee relationships [15]
TR	16	58	7.25	Relationships among New Guinea tribes [16]
VBF	32	356	22.25	University freshman friendship network [17]
FB	32	124	7.75	Online friendship network (Facebook) [18]

Table 4: Summary of the empirical networks used in this study.

References

[1] M. Vickers and S. Chan. *Representing Classroom Social Structure*. Tech. rep. Dataset documentation. Melbourne, Australia: Victoria Institute of Secondary Education, 1981.

[2] R. A. Rossi and N. K. Ahmed. "The Network Data Repository with Interactive Graph Analytics and Visualization". *AAAI*. 2015.

[3] K. Faust. Centrality in affiliation networks. *Social Networks*, 19(2): 157–191, 1997.

[4] S. C. Freeman and L. C. Freeman. *The Networkers Network: A Study of the Impact of a New Communications Medium on Sociometric Structure*. Tech. rep. S46. University of California, Irvine, 1979.

[5] W. W. Zachary. An information flow model for conflict and fission in small groups. *Journal of Anthropological Research*, 33: 452–473, 1977.

[6] J. Gil-Mendieta and S. Schmidt. The political network in Mexico. *Social Networks*, 18: 355–381, 1996.

[7] S. Sundaresan, I. Fischhoff, J. Dushoff, and D. Rubenstein. Network metrics reveal differences in social organization between two fission-fusion species, Grevy's zebra and onager. *Oecologia*, 151: 140–149, 2007.

[8] D. F. Lott. Dominance relations and breeding rate in mature male American bison. *Zeitschrift für Tierpsychologie*, 49(4): 418–432, 1979.

[9] M. W. Schein and M. H. Fohrman. Social dominance relationships in a herd of dairy cattle. *The British Journal of Animal Behaviour*, 3(2): 45–55, 1955.

[10] T. R. Grant. Dominance and association among members of a captive and a free-ranging group of grey kangaroos (*Macropus giganteus*). *Animal Behaviour*, 21(3): 449–456, 1973.

[11] R. L. Breiger, S. A. Boorman, and P. Arabie. An algorithm for clustering relational data with applications to social network analysis and comparison with multidimensional scaling. *Journal of Mathematical Psychology*, 12(3): 328–383, 1975.

[12] C. C. Hass. Social status in female bighorn sheep (*Ovis canadensis*): Expression, development and reproductive correlates. *J. Zool.*, 225(3): 509–523, 1991.

[13] E. G. Schwimmer. "Exchange in the Social Structure of the Orokaiva". PhD thesis. Vancouver: University of British Columbia, 1970.

[14] D. S. Sade. Sociometrics of Macaca mulatta I. Linkages and Cliques in Grooming Matrices. *Folia Primatologica*, 18(3-4): 196–223, 1972.

[15] B. Kapferer. *Strategy and Transaction in an African Factory: African Workers and Indian Management in a Zambian Town*. Manchester University Press, 1972.

[16] K. E. Read. Cultures of the central highlands, New Guinea. *Southwestern Journal of Anthropology*, 10: 1–43, 1954.

[17] G. Van de Bunt, M. Van Duijn, and T. Snijders. Friendship networks through time: An actor-oriented statistical network model. *Computational and Mathematical Organization Theory*, 5: 167–192, 1999.

[18] M. Magnani, B. Micenkova, and L. Rossi. *Combinatorial Analysis of Multiple Networks*. arXiv:1303.4986. 2013.

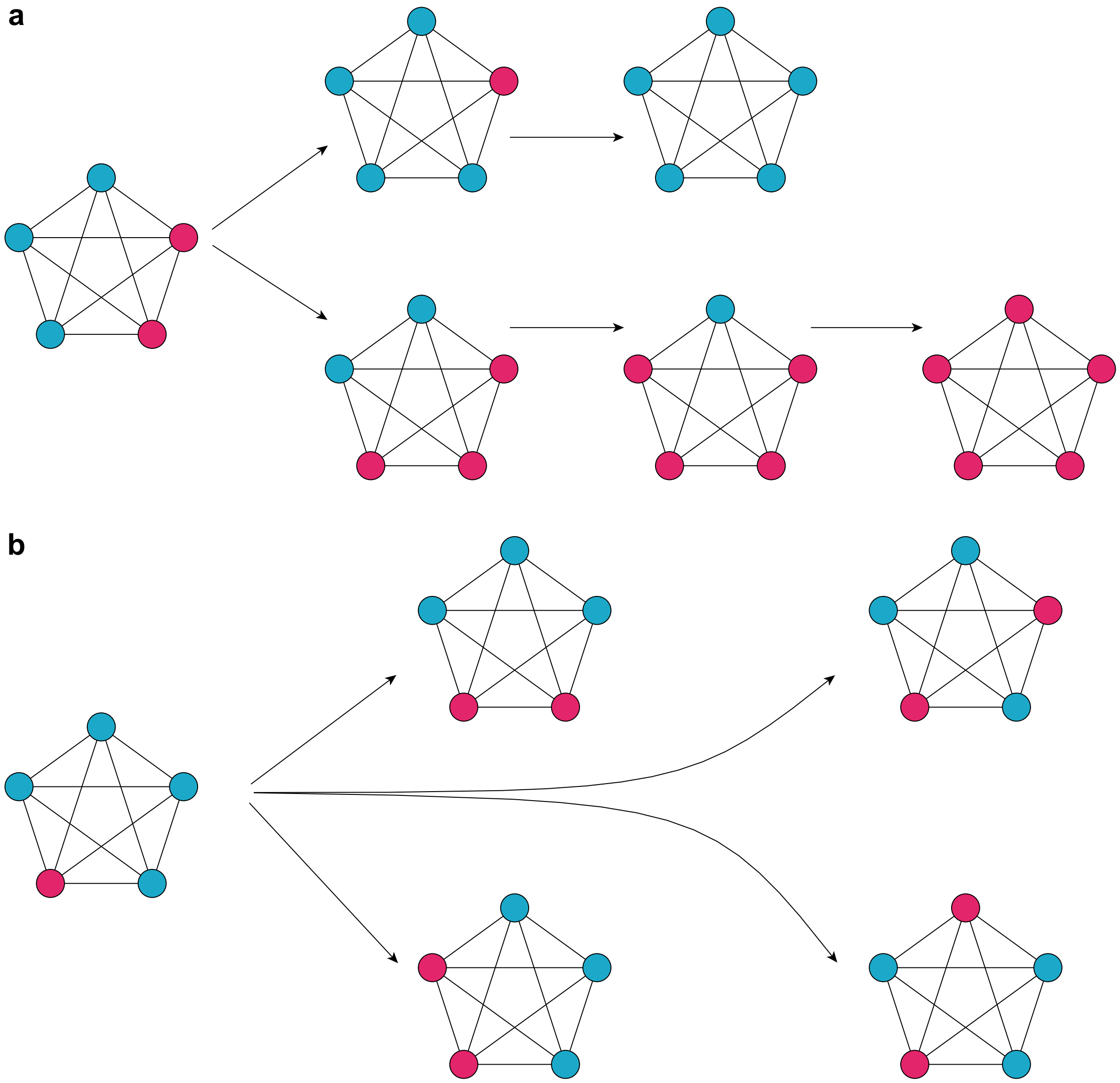

Figure S1: Comparison between coordination game and anti-coordination game. Panel **a** and **b** correspond to coordinating and anti-coordination game respectively. Parameter values: behavioral switching threshold $\tau = 0.37$.

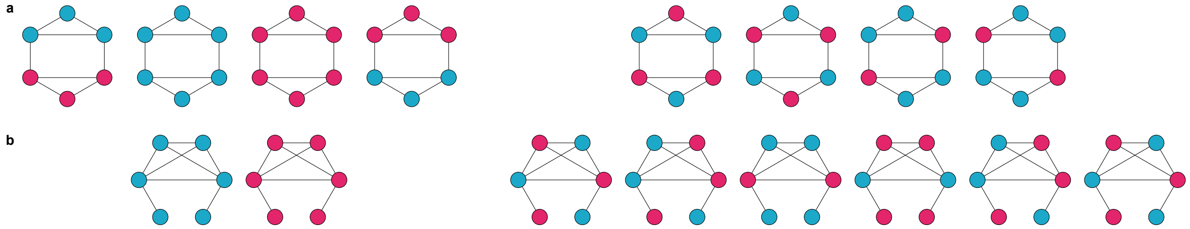

Figure S2: Example of network with high clustering coefficient. Panel **a** and **b** correspond to networks with low and high clustering coefficients respectively. Parameter values: behavioral switching threshold $\tau = 0.37$.

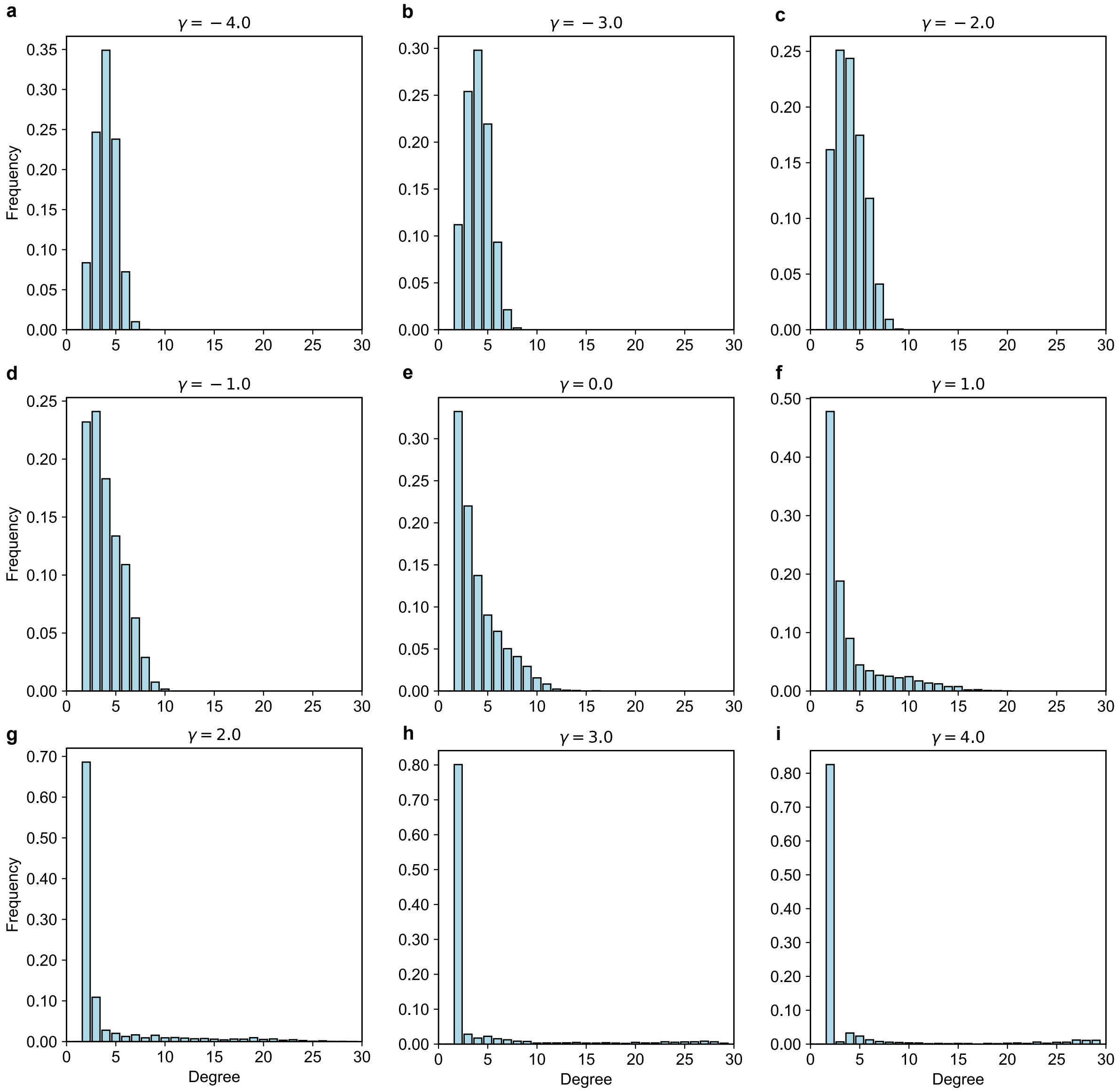

Figure S3: Degree distribution of networks with different connection kernel. In each panel, we take 100 networks to calculate the average degree distribution. Parameters: network size $N = 30$, average degree $\bar{k} = 4.0$

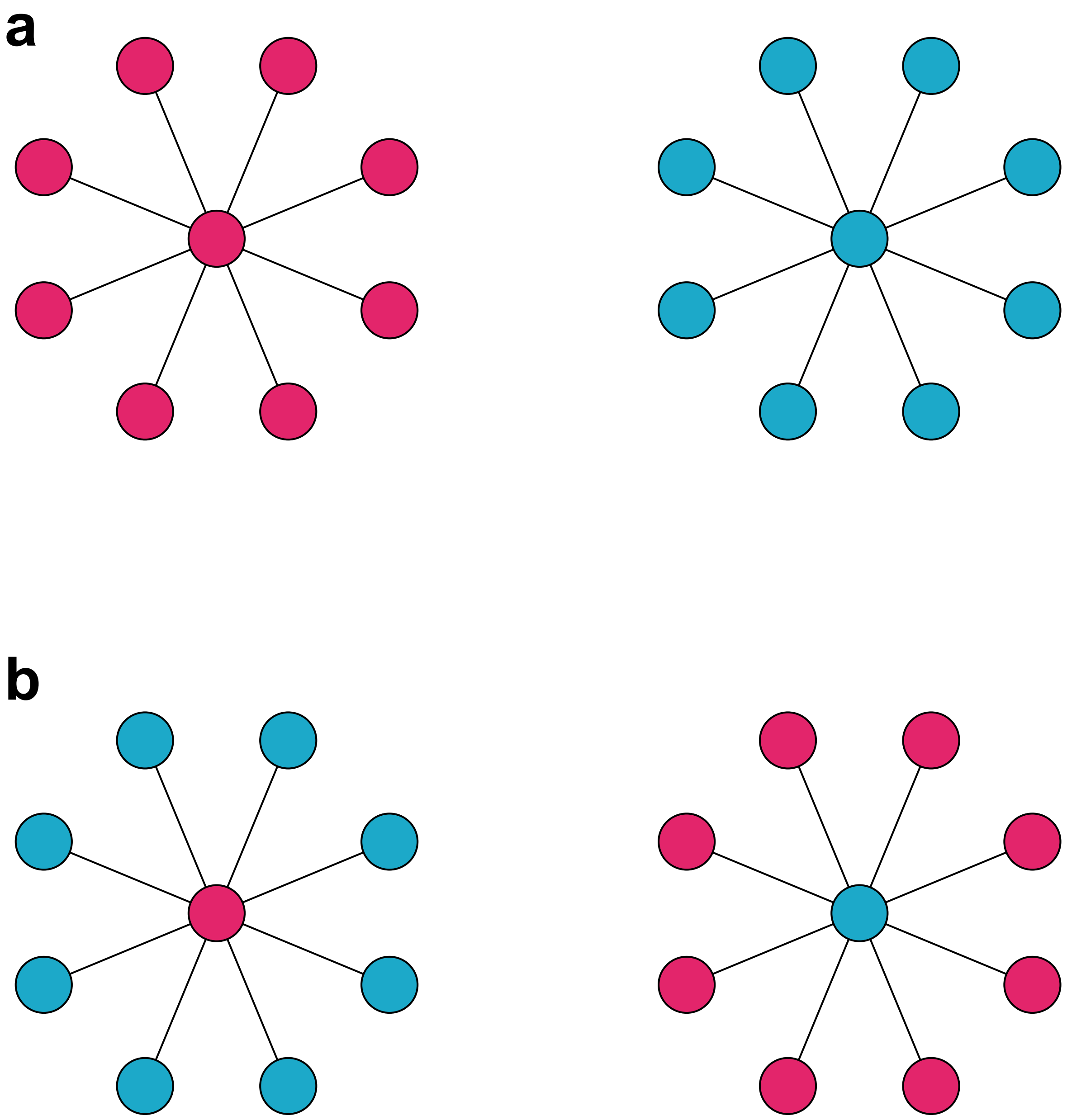

Figure S4: Example of heterogeneous network. Panel **a** and **b** correspond to coordinating and anti-coordinating game respectively. Parameter values: behavioral switching threshold $\tau = 0.37$.

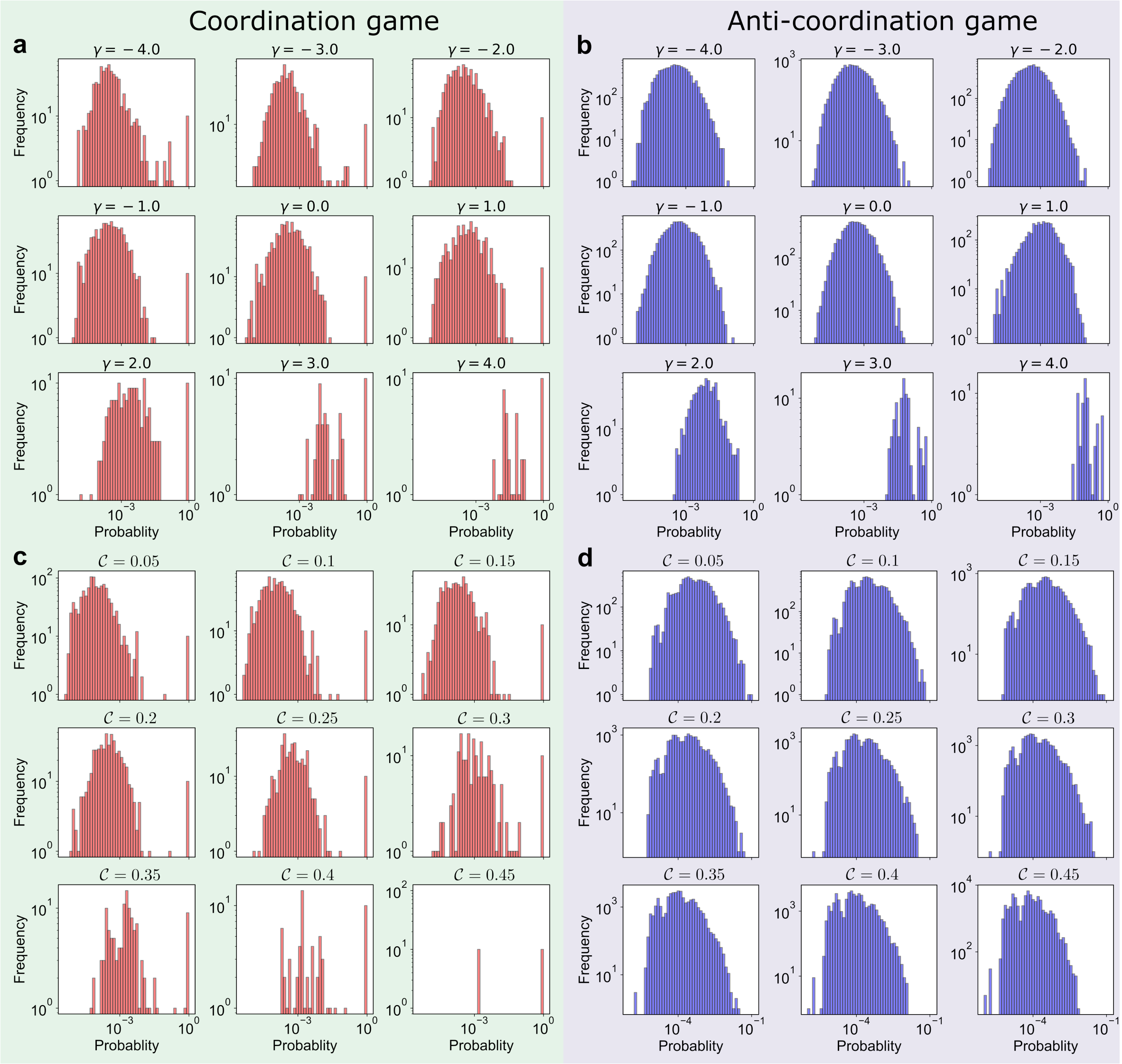

Figure S5: There exists equilibrium states with high probabilities of being reached in any coordination game and anti-coordination game with significant node degree distribution heterogeneity. We analyzed the arrival probability of each equilibrium state. The coordinating(**ac**) and anti-coordinating(**bd**) games are both considered. In each subfigure of panel **a-d**, the horizontal axis represents the arrival probability, while the vertical axis indicates the number of equilibrium states whose reaching probabilities fall within the intervals defined on the horizontal axis. **ab** and **cd** indicate the influence of the clustering coefficient($\mathscr{C}$) and the node degree distribution on the probability. Each panel is the result of 30000000 simulations. Parameter values: network size $|\mathscr{N}| = 30$, average degree $\bar{k} = 4$.

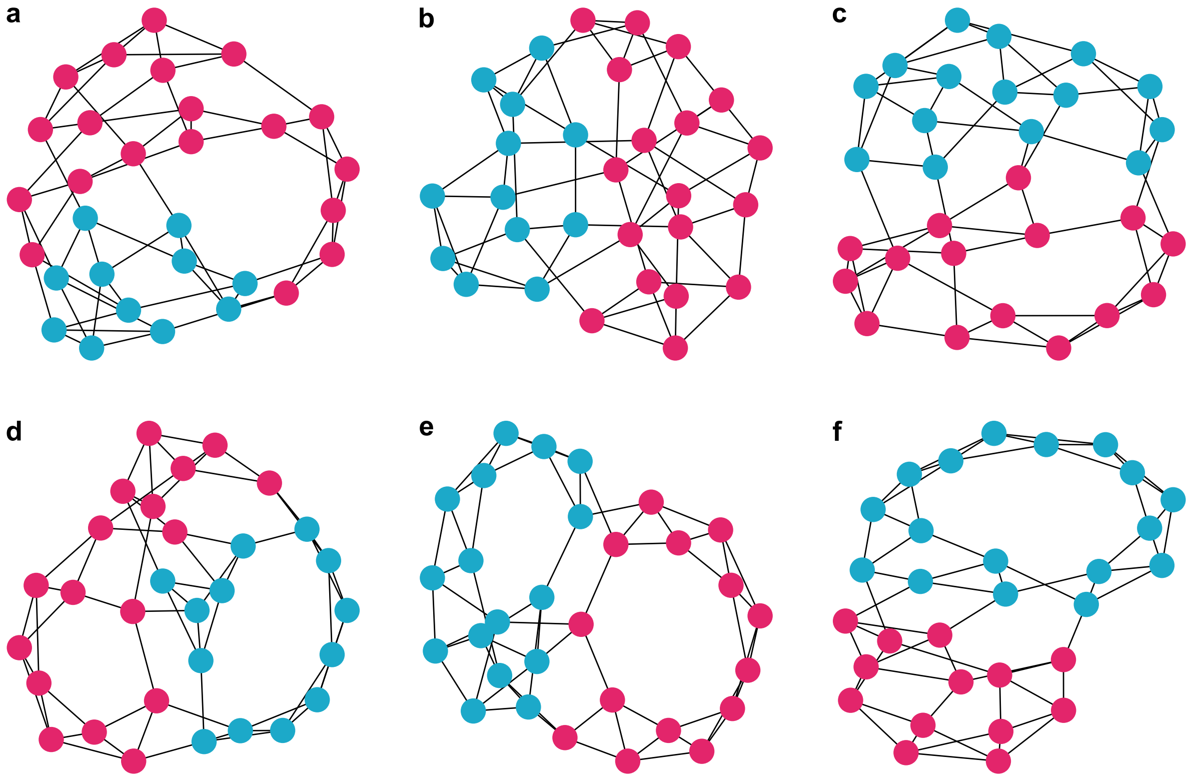

Figure S6: Strategies coexist in networks with local clusters. The clustering coefficient of networks in panel **a-c** and **e-f** are 0.25 and 0.35 respectively. Parameters: network size $N = 30$, behavioral switching threshold $\tau = 0.37$, average degree $\bar{k} = 4$

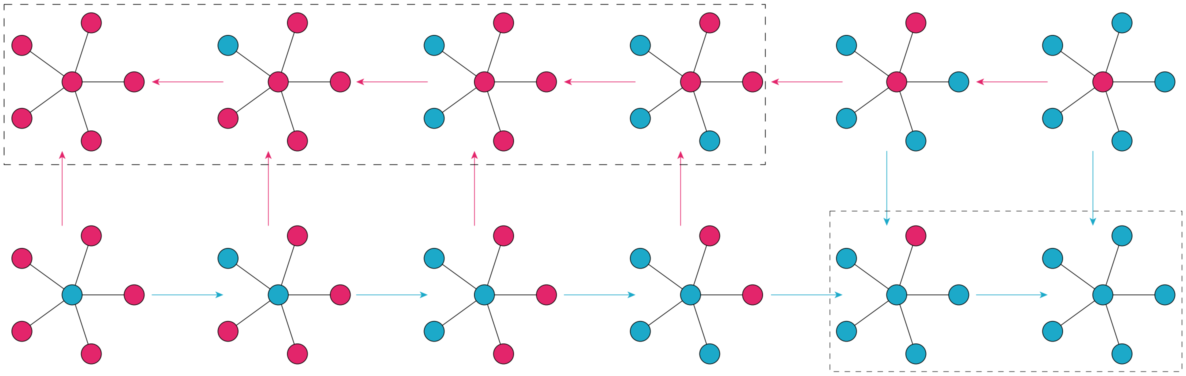

Figure S7: State transition graph of star network in the coordination game. The average equilibrium time of this system is 2.625. Parameter values: network size $N = 6$, behavioral switching threshold $\tau = 0.37$.

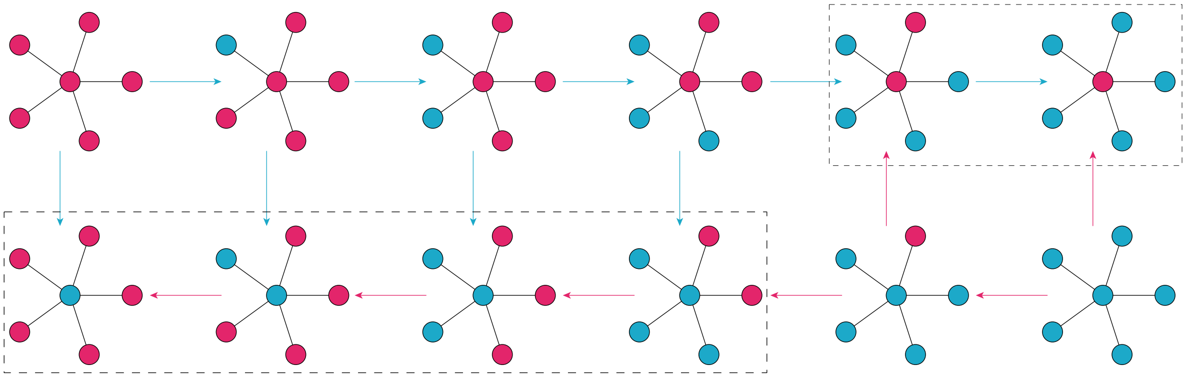

Figure S8: State transition graph of star network in the anti-coordination game. The average equilibrium time of this system is 2.625. Parameter values: network size $N = 6$, behavioral switching threshold $\tau = 0.37$.

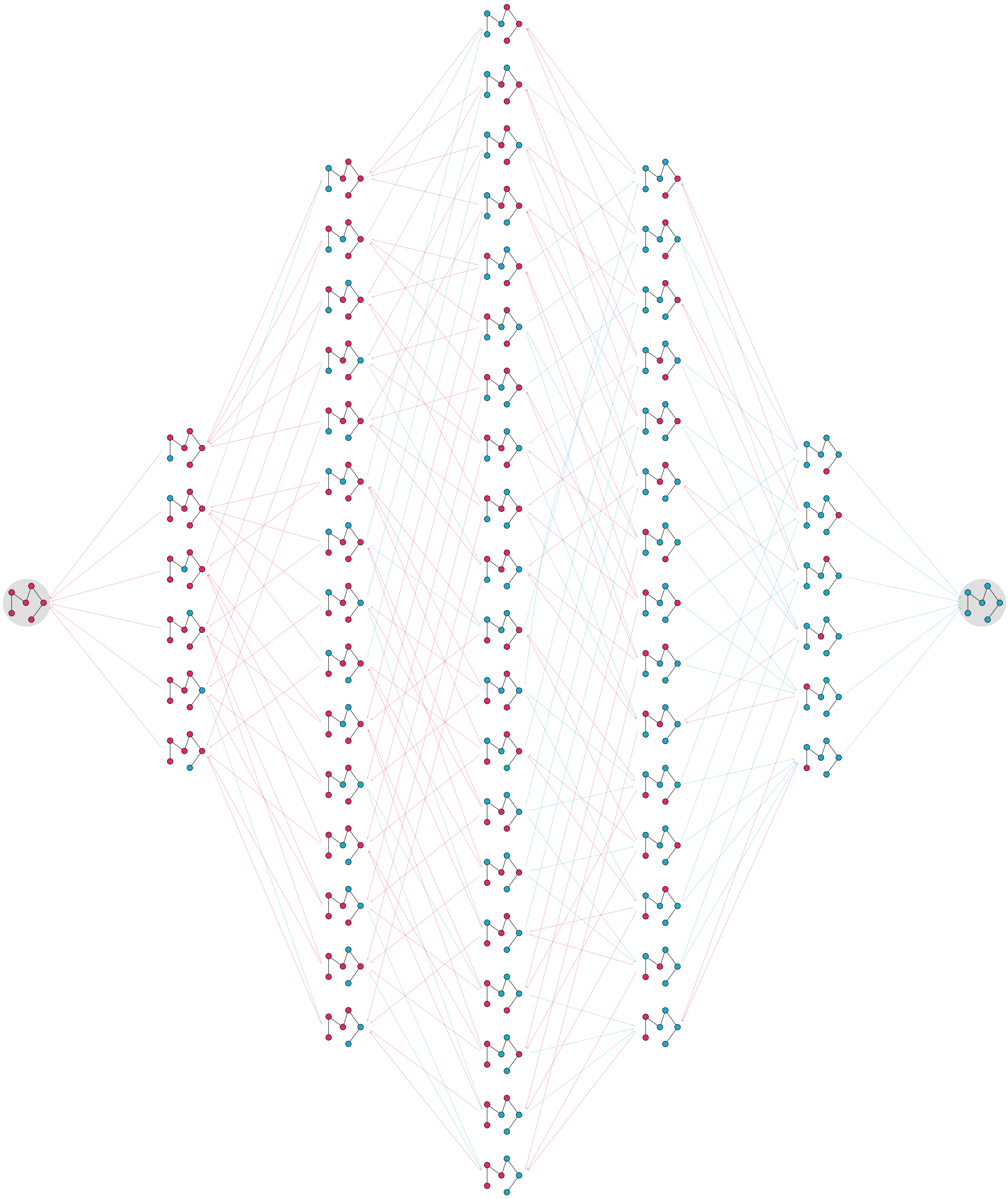

Figure S9: State transition graph of chain network in the coordination game. The average equilibrium time of this system is 3.325. Parameter values: network size $N = 6$, behavioral switching threshold $\tau = 0.37$.

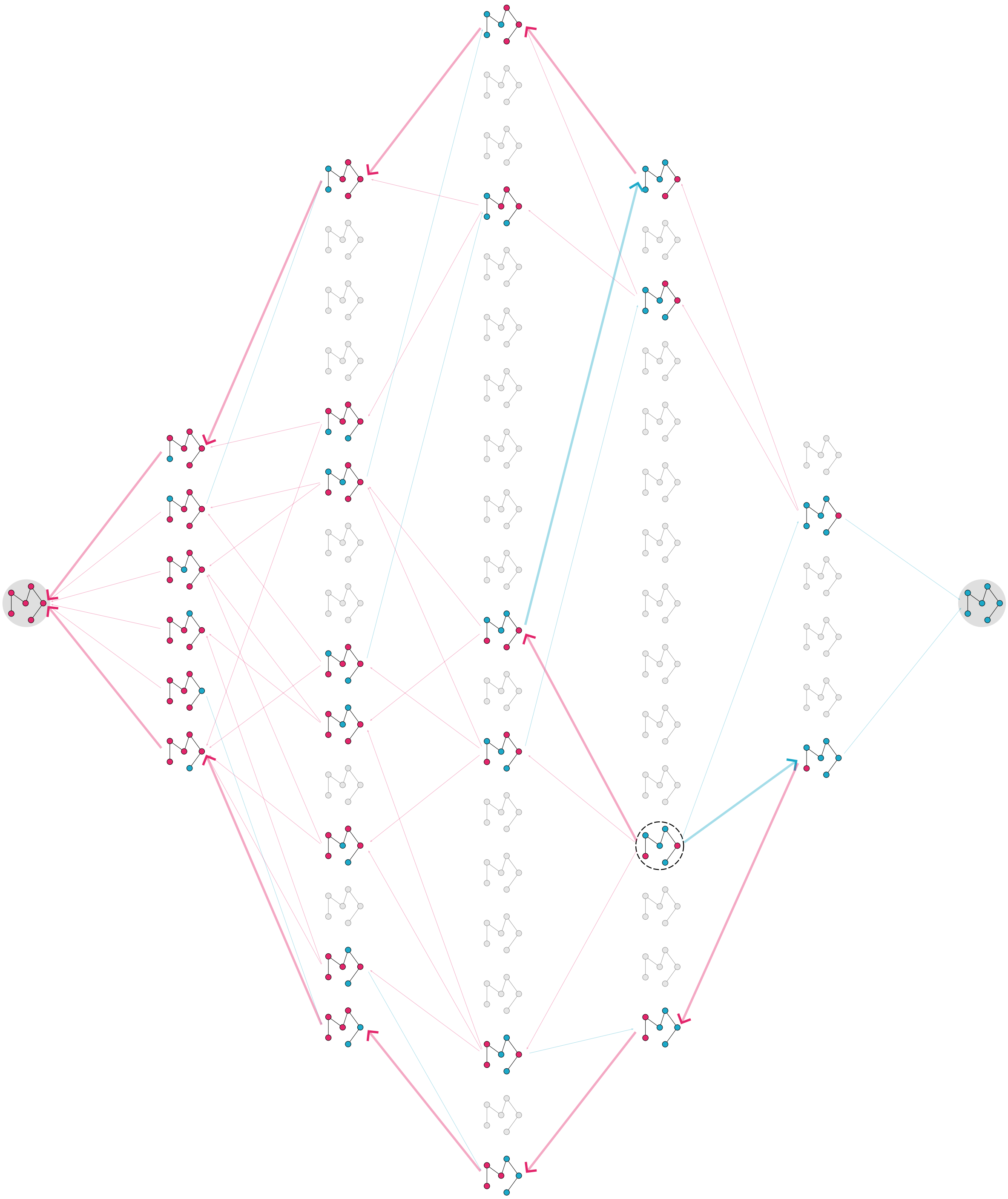

Figure S10: Example of the evolution process of chain network in the coordination game. Parameter values: network size $N = 6$, behavioral switching threshold $\tau = 0.37$.

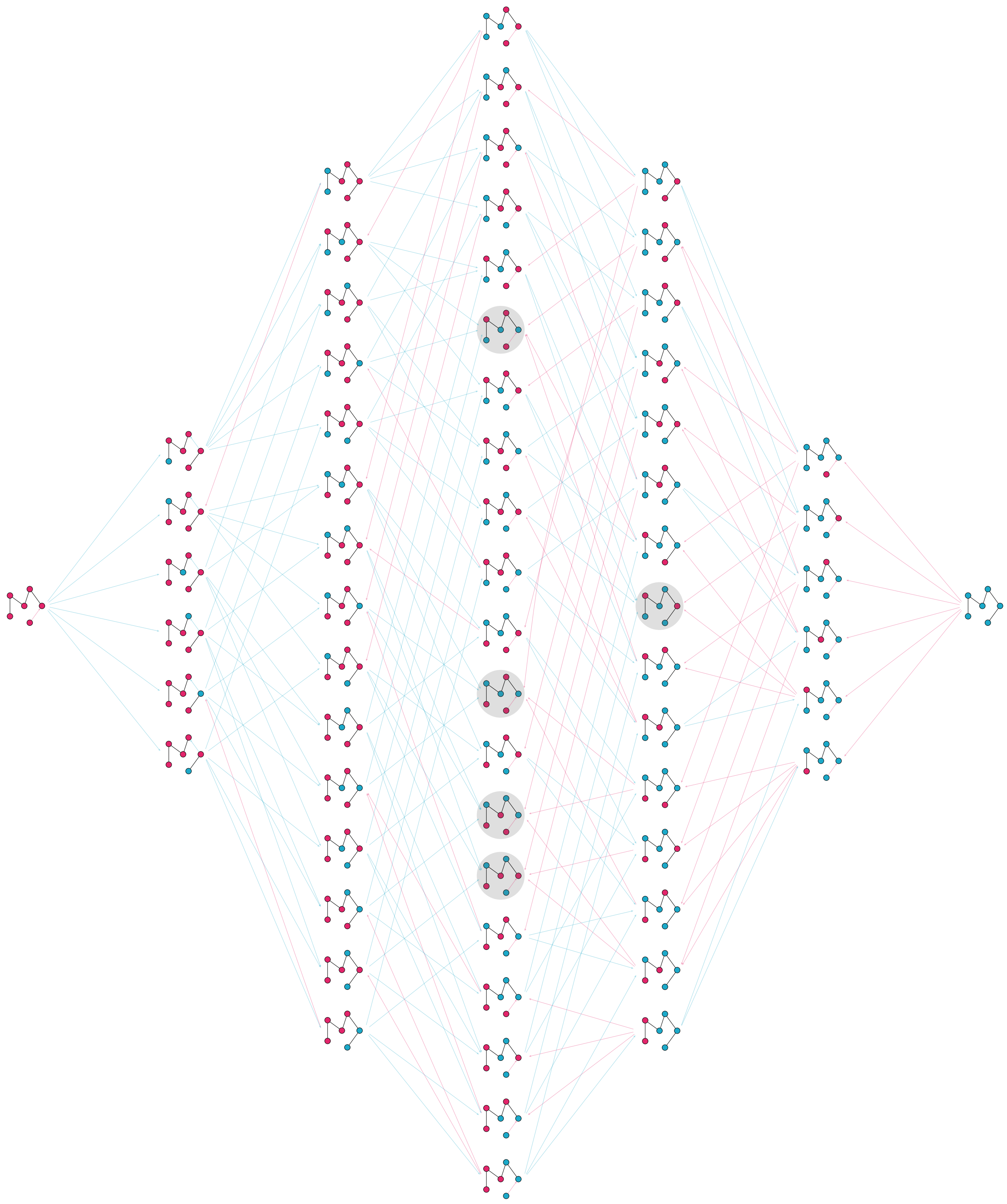

Figure S11: State transition graph of chain network in the anti-coordination game. The average equilibrium time of this system is 2.050. Parameter values: network size $N = 6$, behavioral switching threshold $\tau = 0.37$.

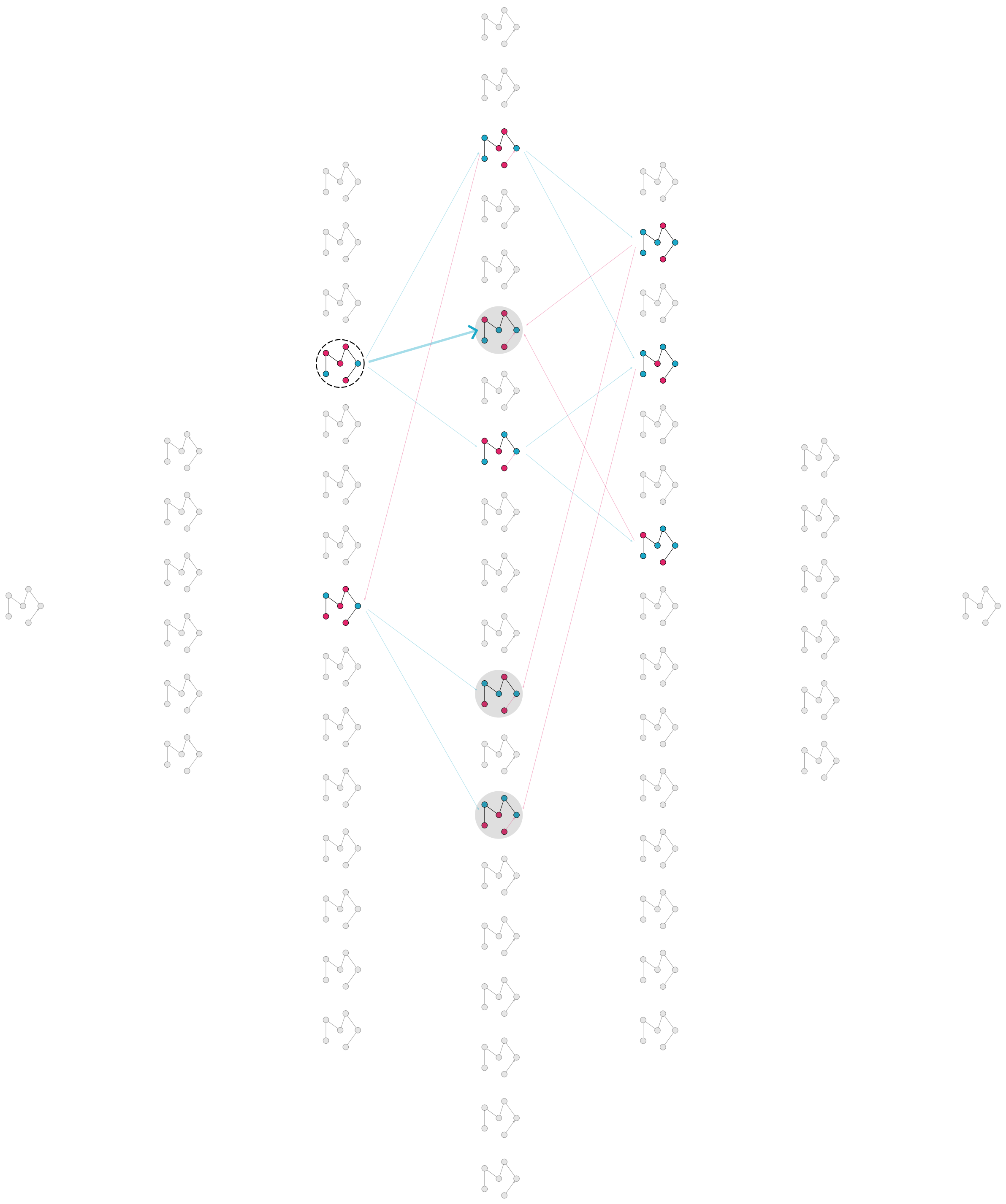

Figure S12: Example of the evolution process of chain network in the anti-coordination game. Parameter values: network size $N = 6$, behavioral switching threshold $\tau = 0.37$.

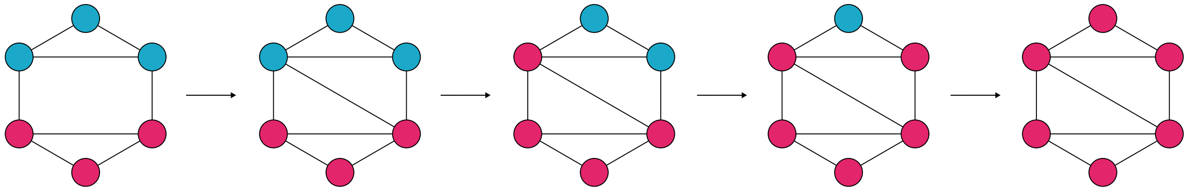

Figure S13: Adding one new edge can make the favored strategy dominate the whole system. Parameters: behavioral switching threshold $\tau = 0.37$

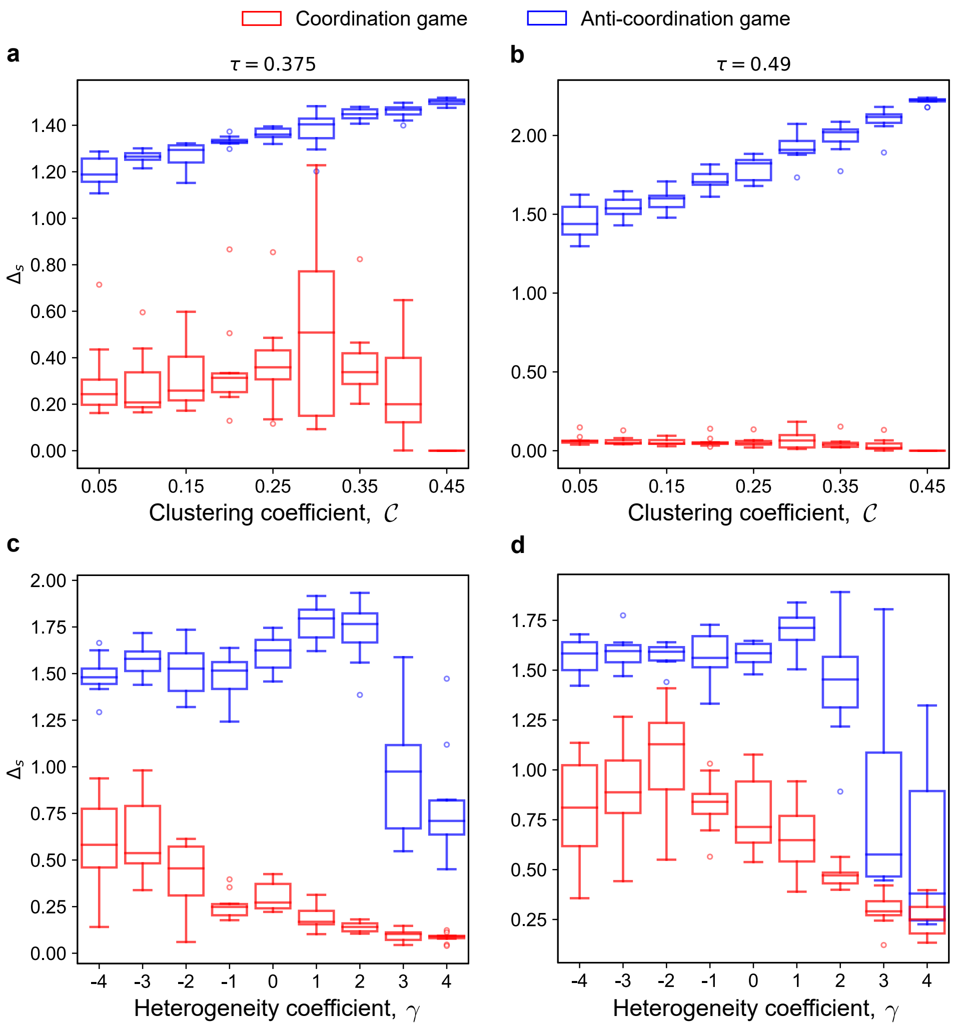

Figure S14: Robustness analysis with adding 5 new edges. Each red (respectively blue) box represents the average strategy changing under the coordination game (respectively under the anti-coordination game) in 50 networks, showing the median, quartiles, and outliers. We simulate each network of 2×10^8 times, where five edges are randomly added to each simulation. Panel **ab** displays the variations in the average number of strategy switches as the clustering coefficients change, and panel **cd** depicts how the average number of strategy switches evolves with the increase in degree heterogeneity.

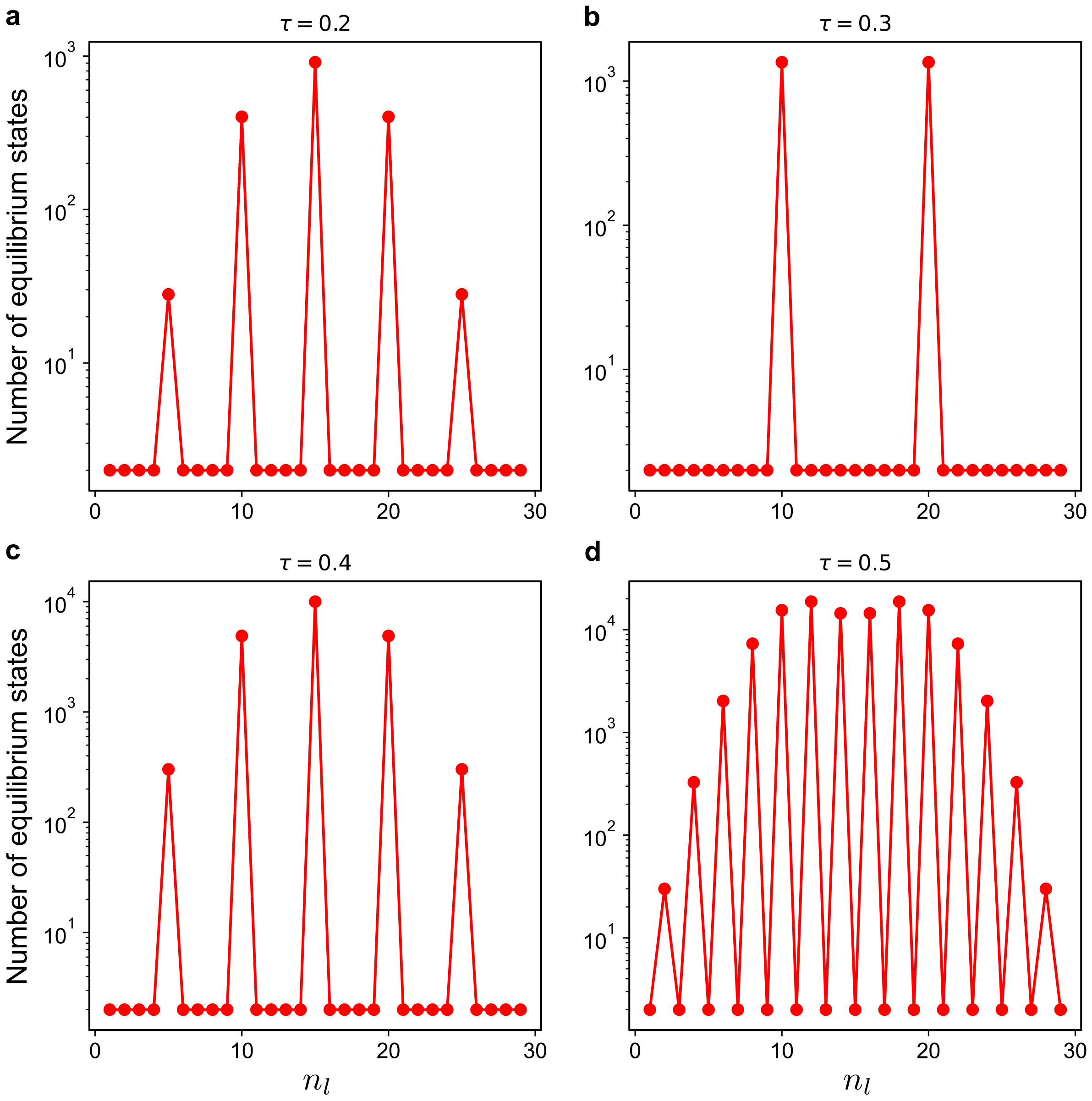

Figure S15: Number of equilibrium states of the bipartite graph could be extremely high. Presented is how the number of equilibrium states changes with the increase of left-side nodes. The total number of nodes remains unchanged. Parameters: network size $N = 30$

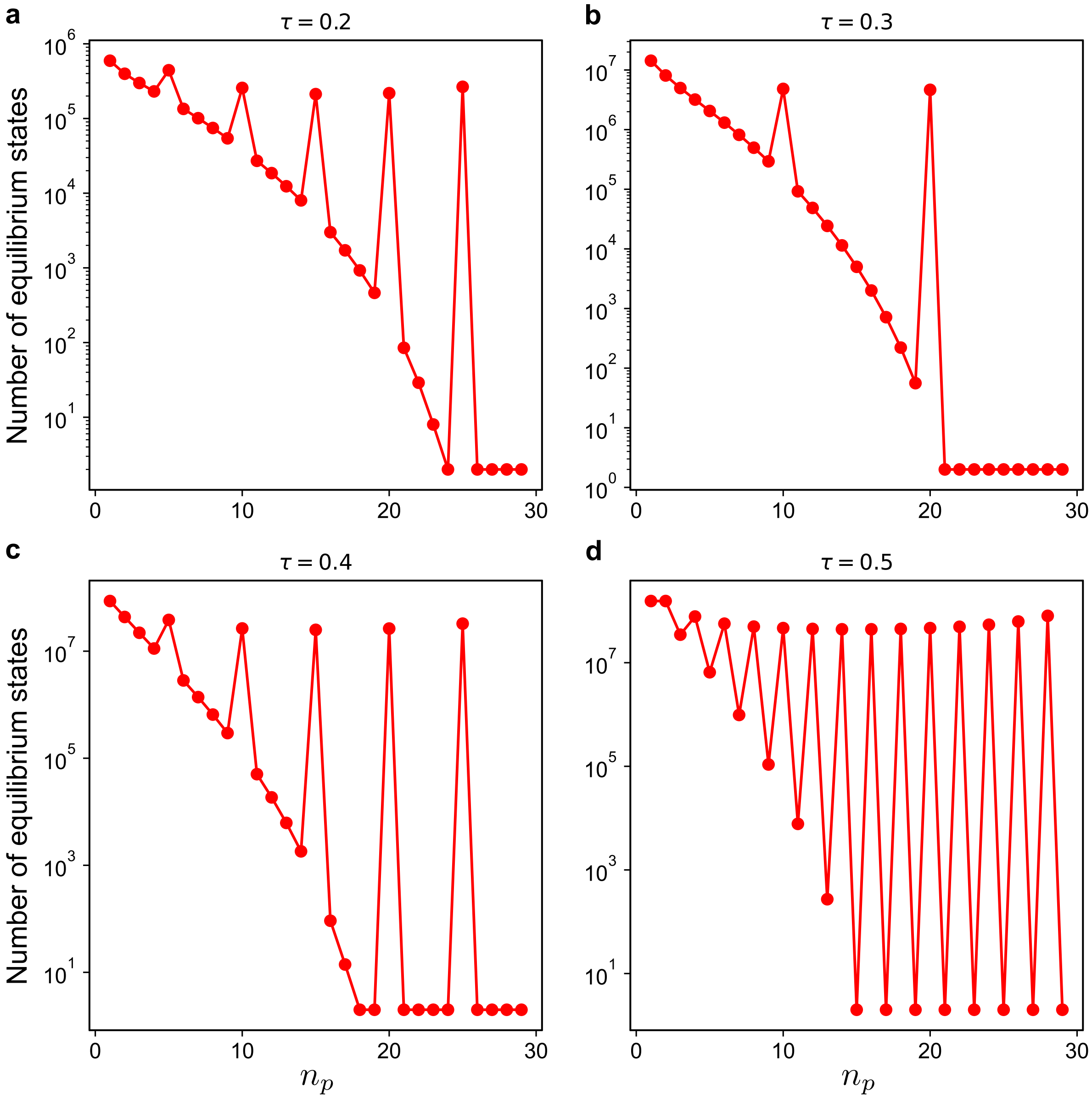

Figure S16: Number of equilibrium states of the rich-club network could be extremely high. Presented is how the number of equilibrium states changes with the increase of poor nodes. The total number of nodes remains unchanged. Parameters: network size $N = 30$

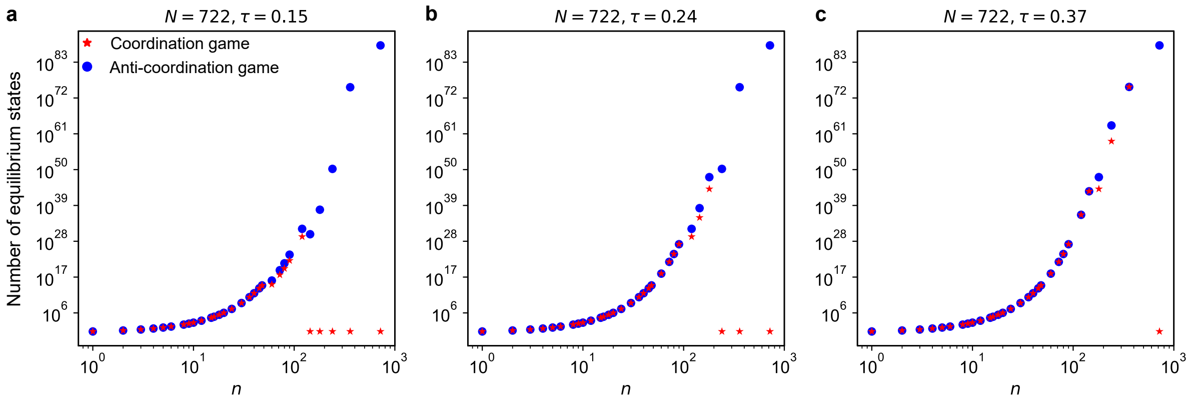

Figure S17: The number of equilibrium states rises to a peak before decreasing in coordination games and increases with the increase of the average eccentricities of the network in anti-coordination games.

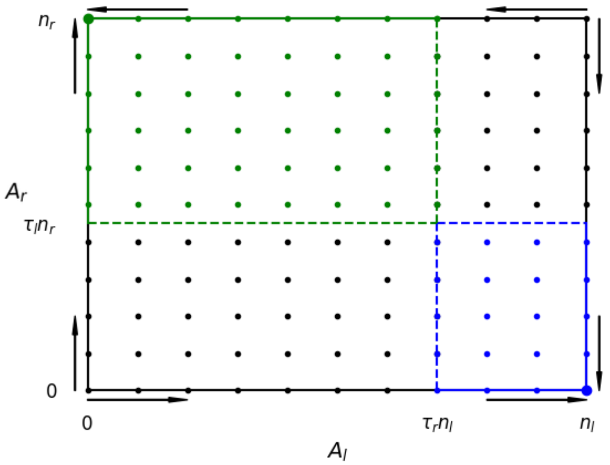

Figure S18: The influence of A-individuals on the equilibrium states

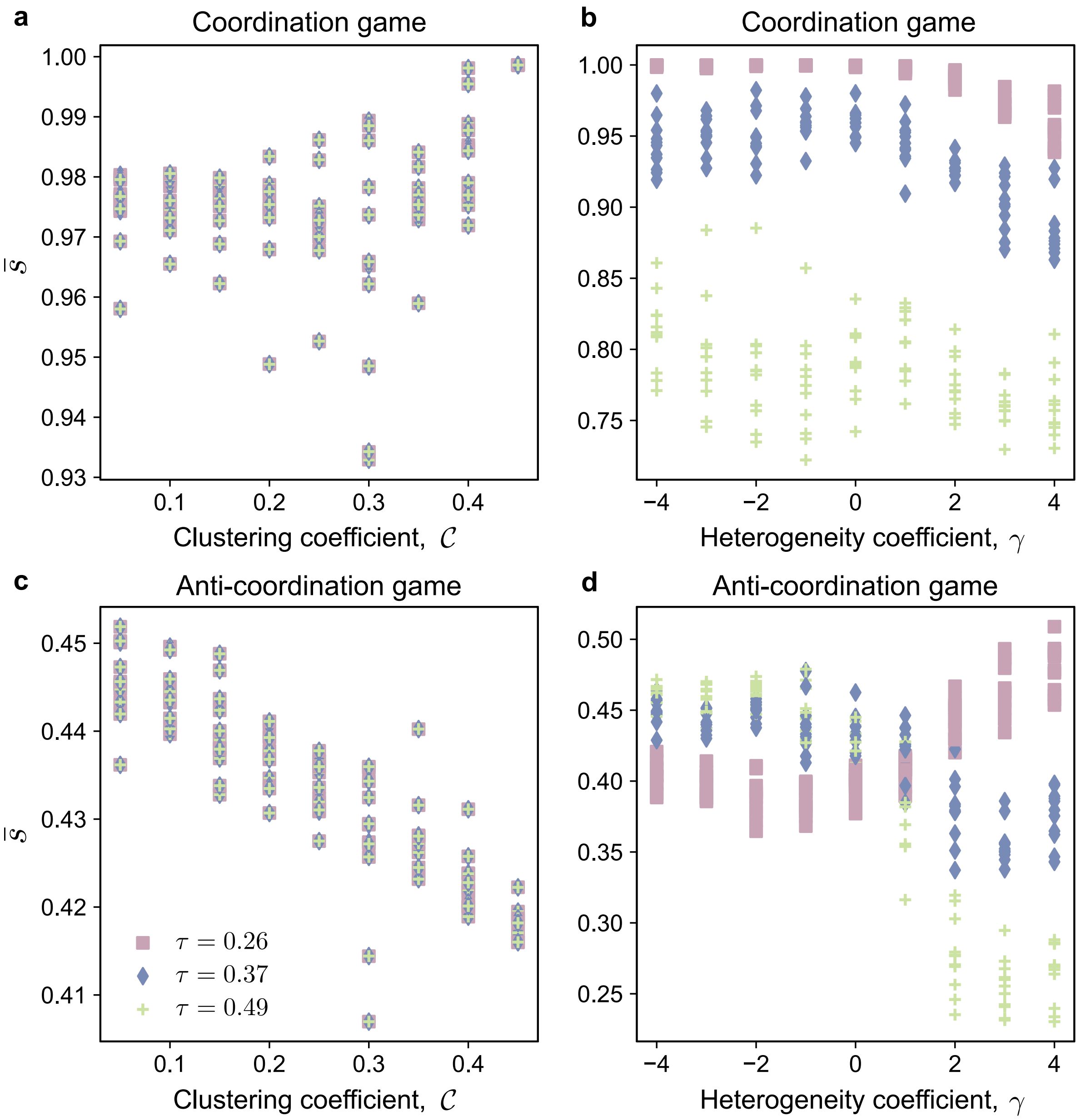

Figure S19: The average frequency of strategy A at different τ values. Here each marker is the average frequency of strategy A in a network. Panel **ac** illustrates the results in 4-regular networks with different clustering coefficients, while panel **bd** shows the result in networks with different degree distribution heterogeneity. The results in 4-regular networks remain unchanged at different τ values because the three values are in the interval of $(0.25, 0.5)$ and any value of τ in this interval has the same effect on the dynamics of the 4-regular network according to Eq. 2 and Eq. 3.

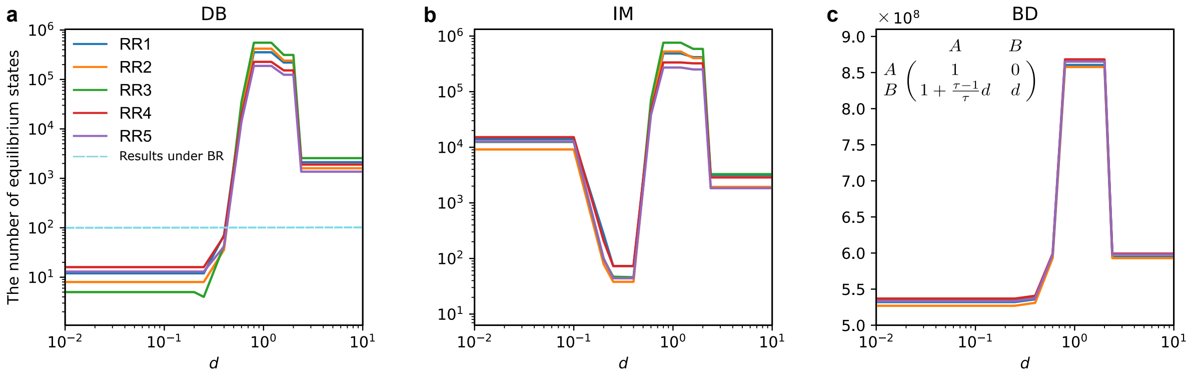

Figure S20: Results under DB/IM/BD updating are sensitive to the payoff value. Here we compare the death-birth, imitation and death-birth learning rules in **a-c**. Different from the results under best-response dynamics, the number of equilibrium states under DB/IM/BD updating changes with the variation of d even if the value of τ is constant. Parameter values: network size $N = 30$ and average degree $\bar{k} = 4$.

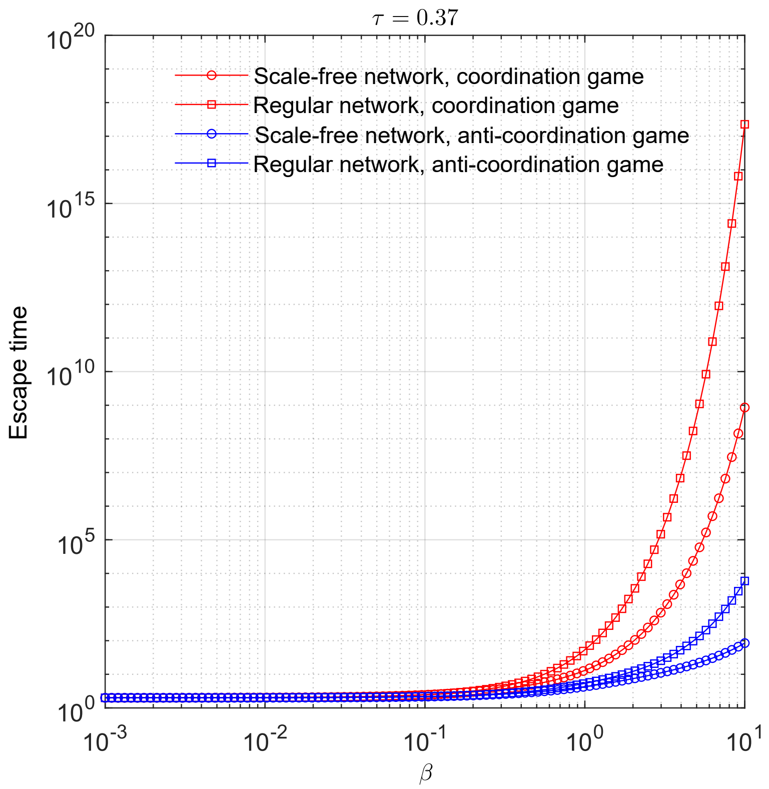

Figure S21: Escape time as the players' rationality level increases (noise level decreases).

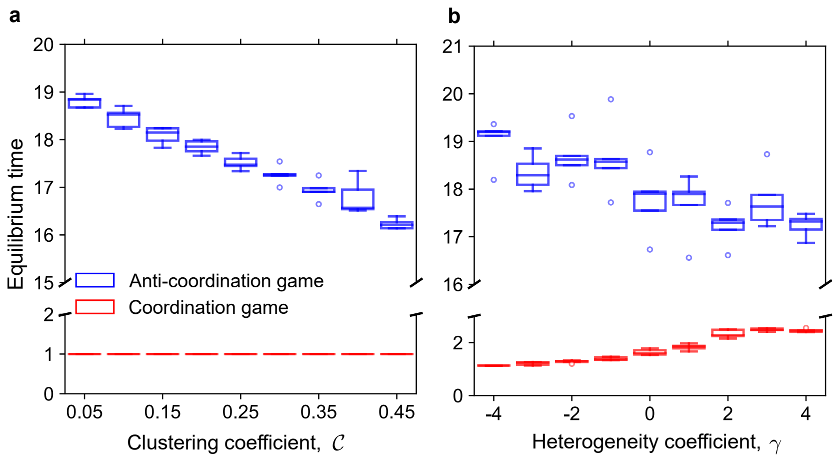

Figure S22: Equilibrium time when only one A-player at the initial state. Parameters: network size $N = 30$, behavioral switching threshold $\tau = 0.37$

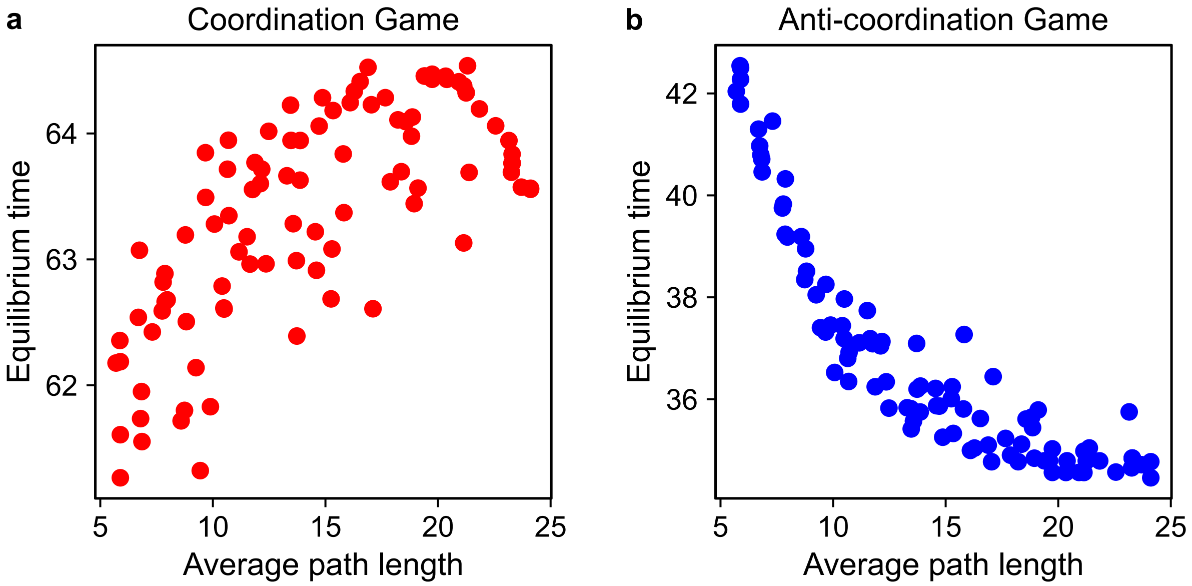

Figure S23: Average equilibrium time grows (shrinks) as the average path length increases under coordination (anti-coordination) games in larger networks. The average equilibrium time of different networks is shown as a function of the average path length. Each dot in panels **a** and **b** represents the average equilibrium time for one network starting from random initial states. The simulation is repeated 3×10^7 times for each network. Parameter values: network size $N = 100$ and average degree $\bar{k} = 4$.

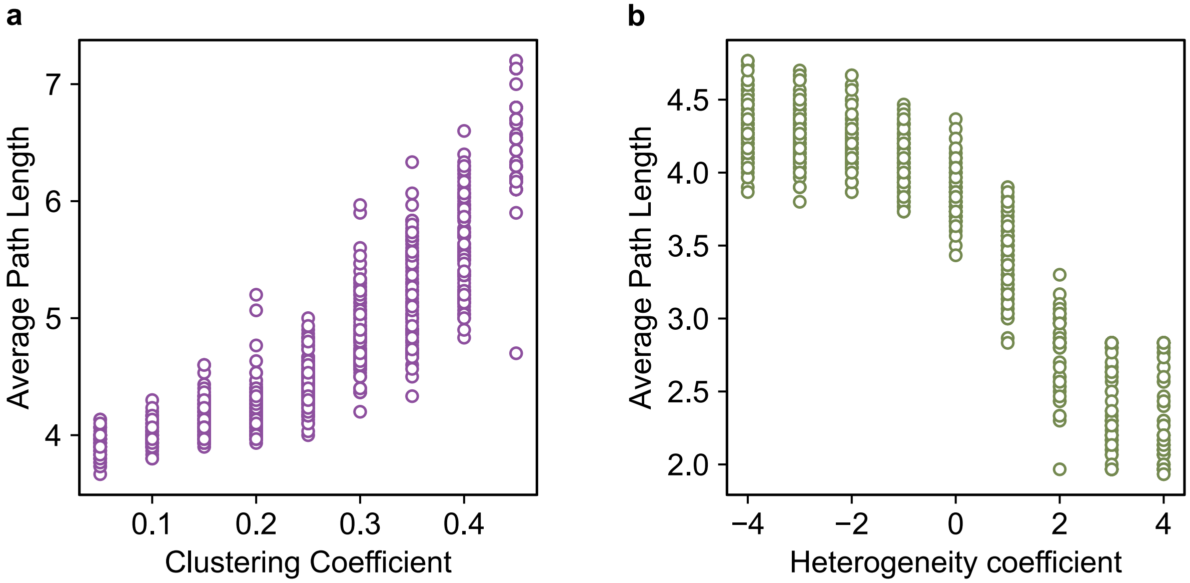

Figure S24: Average path length increases as the clustering coefficient increases, while decreases as the heterogeneity coefficient increases.
